# Supplementary material for: A unifying modelling of multiple land degradation pathways in Europe
Source: Nat Commun. 2024 May 8;15:3862. doi: 10.1038/s41467-024-48252-x (PMC11079025; doi:10.1038/s41467-024-48252-x)
Supplement: Supplementary file 1 — Supplementary information [file 41467_2024_48252_MOESM1_ESM.pdf]

# Supplementary Information

## A unifying modelling of multiple land degradation pathways in Europe

Remus PRĂVĂLIE [1,2,3 *], Pasquale BORRELLI [4,5], Panos PANAGOS [6], Cristiano BALLABIO [6], Emanuele LUGATO [6], Adrian CHAPPELL [7], Gonzalo MIGUEZ-MACHO [8], Federico MAGGI [9], Jian PENG [10], Mihai NICULIȚĂ [11], Bogdan ROȘCA [12], Cristian PATRICHE [12], Monica DUMITRAȘCU [13], Georgeta BANDOC [1,3], Ion-Andrei NITA [14], Marius-Victor BIRSAN [13]

[1] University of Bucharest, Faculty of Geography, 1 Nicolae Bălcescu Street, 010041, Bucharest, Romania
[2] University of Bucharest, Research Institute of the University of Bucharest (ICUB), 90–92 Panduri Street, 050663, Bucharest, Romania
[3] Academy of Romanian Scientists, 54 Splaiul Independentei Street, 050094, Bucharest, Romania
[4] Department of Environmental Sciences, Environmental Geosciences, University of Basel, Basel, Switzerland
[5] Department of Science, Roma Tre University, Rome, Italy
[6] European Commission, Joint Research Centre (JRC), Ispra, Italy
[7] School of Earth and Environmental Sciences, Cardiff University, Wales, United Kingdom
[8] CRETUS, Non-Linear Physics Group, Faculty of Physics, Universidade de Santiago de Compostela, Galicia, Spain
[9] Environmental Engineering, School of Civil Engineering, The University of Sydney, Sydney, New South Wales, Australia
[10] Laboratory for Earth Surface Processes, Ministry of Education, College of Urban and Environmental Sciences, Peking University, Beijing, China
[11] Alexandru Ioan Cuza University, Faculty of Geography and Geology, Department of Geography, 20A Carol I Street, 700506, Iași, Romania
[12] Romanian Academy, Iași Divison, Geography Department, 8 Carol I Street, 700505, Iași, Romania
[13] Institute of Geography, Romanian Academy, 12 Dimitrie Racoviță Street, 023993, Bucharest, Romania
[14] Visual Flow, 140 Aurel Vlaicu, 020099, Bucharest, Romania

* Corresponding author (pravalie_remus@yahoo.com)

**This PDF file contains:**
Supplementary Figures (1–4)
Supplementary Tables (1–13)

## Supplementary Figures

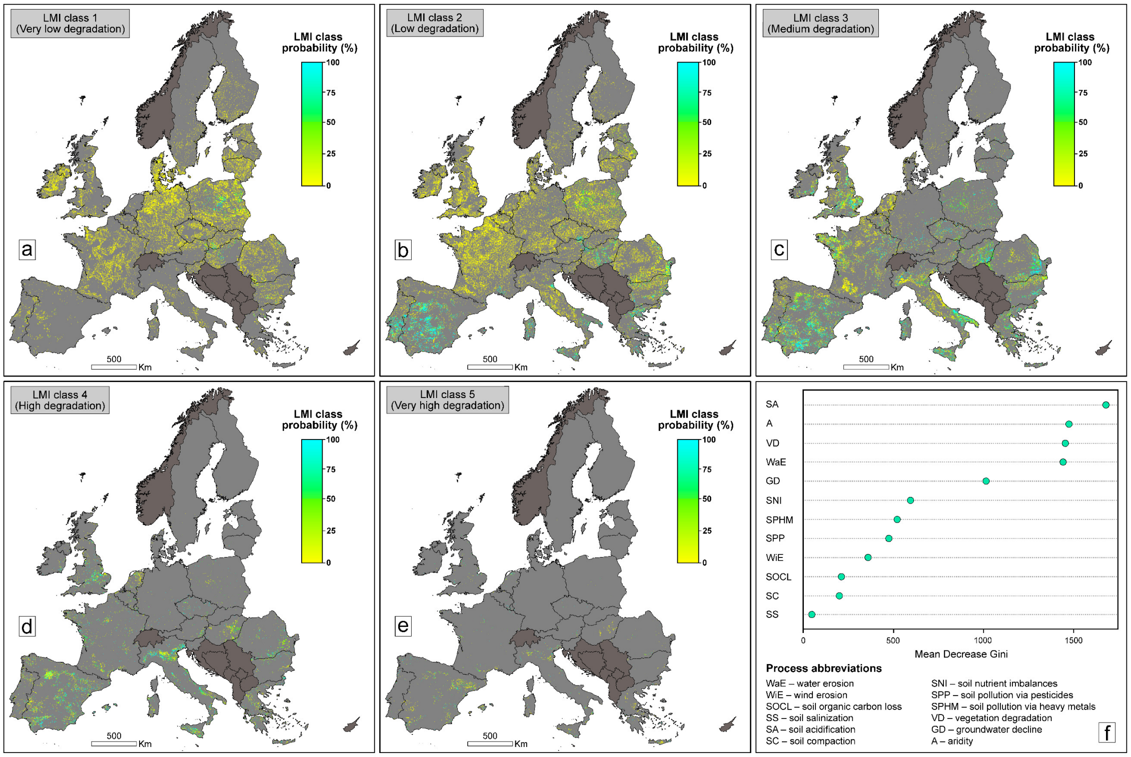


**Supplementary Fig. 1.** Uncertainty and sensitivity of LMI results in Europe. **a–e**, Spatial distribution of uncertainty presented as probability (%) for LMI classes 1–5. **f**, Covariates used in applying the RF model, in order to predict uncertainties of LMI classes and provide a sensitivity analysis. Notes: probability (a–e) ranges from 0 (low) to 100 (high), and was obtained by applying a RF classification model (1,000 decision trees and 20 repetitions); covariates were used in the RF classification model for predicting with which probability a pixel falls within a given LMI class; covariate importance is expressed by the Mean Decrease Gini, which is a measure of how each input variable contributes in the prediction of LMI classes; the higher the value of mean decrease Gini coefficient, the higher the importance of the variable in the model is, thus reflecting the sensitivity of each variable for predicting LMI classes; RF means Random Forest, while LMI is the acronym for Land Multi-degradation Index.

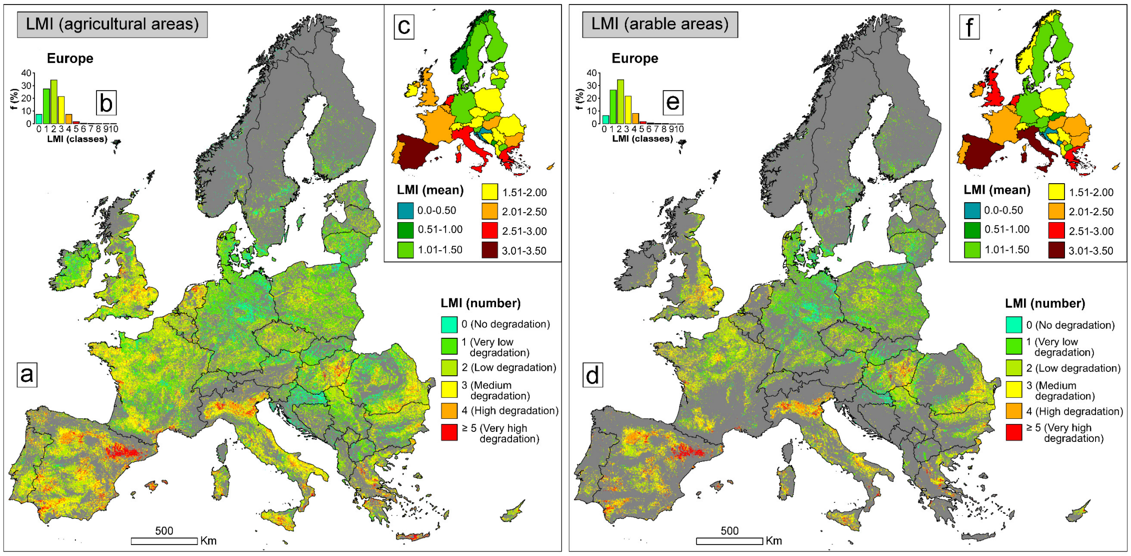


**Supplementary Fig. 2.** Spatial pattern of land multi-degradation in Europe (all 40 investigated countries). **a**, Spatial distribution of LMI values (number of co-occurring processes) in agricultural landscapes. **b**, Histogram of LMI values for European agricultural lands. **c**, Average number of co-occurring processes in agricultural environments of continental countries. **d**, Spatial distribution of LMI values (number of co-occurring processes) in arable landscapes. **e**, Histogram of LMI values for European arable lands. **f**, Average number of co-occurring processes in arable environments of continental countries. Notes: light grey highlights non-agricultural/non-arable lands; compared to Fig. 2, the LMI values are spatialized here for all analysed states, considering that in some cases with incomplete data (Norway, Switzerland, Balkan countries, Cyprus and Malta) we still used a large number of process databases (9–10 input layers out of 12) in computing LMI (see *Methods*); consequently, LMI still has the potential to provide a good/very good picture of land degradation in these particular countries, even if their LMI values should be interpreted with some caution; LMI is the acronym for Land Multi-degradation Index; the source data for the graphs in this figure are provided as a Source Data file.

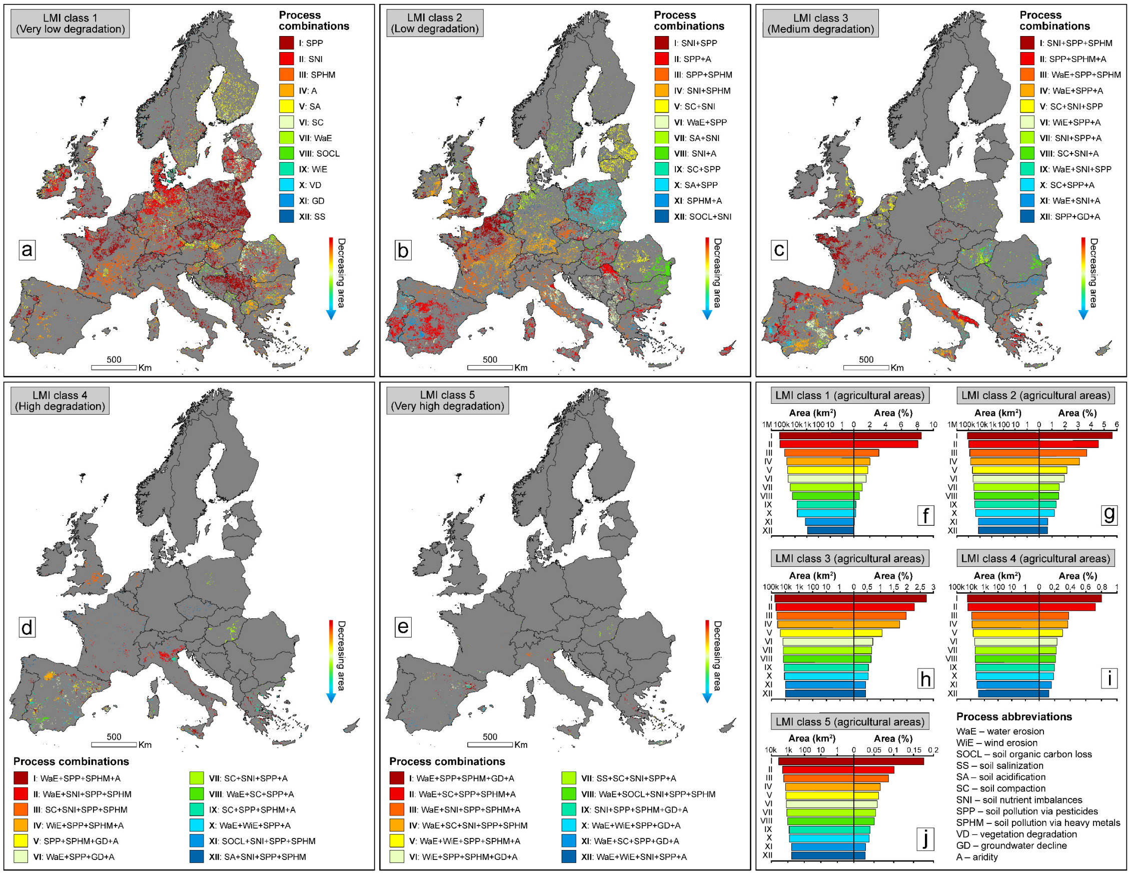


**Supplementary Fig. 3.** Spatial pattern of interacting convergent processes in agricultural environments of Europe (all 40 investigated countries). **a–e**, Spatial distribution of the dominant (most frequent) co-occurring process types (combinations) in LMI agricultural classes 1 (a), 2 (b), 3 (c), 4 (d) and 5 (e). **f–j**, Absolute and percentage-based (% of the total continental agricultural lands) spatial footprint of the dominant co-occurring process types in LMI agricultural classes 1 (f), 2 (g), 3 (h), 4 (i) and 5 (j). Notes: in order to simplify the mapping of process combinations (which are very numerous for each LMI class, except for class 1), in this figure the twelve most important types of co-occurring processes in Europe were selected, which, in terms of area, cumulatively account for at least 50% of all LMI class combinations; to better highlight the mapped process combinations, pixel size was increased to 5 km × 5 km, but the quantification of process combination areas (in km$^2$ and %) was done using the original data resolution of 500 m × 500 m; compared to Fig. 3, the process combinations are spatialized here for all analysed states, considering that in some cases with incomplete data (Norway, Switzerland, Balkan countries, Cyprus and Malta) we still used a large number of process databases (9–10 input layers out of 12) in computing LMI (see *Methods*); consequently, LMI still has the potential to provide a good/very good picture of land degradation in these particular countries, even if their LMI combinations should be interpreted with some caution; LMI is the acronym for Land Multi-degradation Index; the source data for the graphs in this figure are provided as a Source Data file.

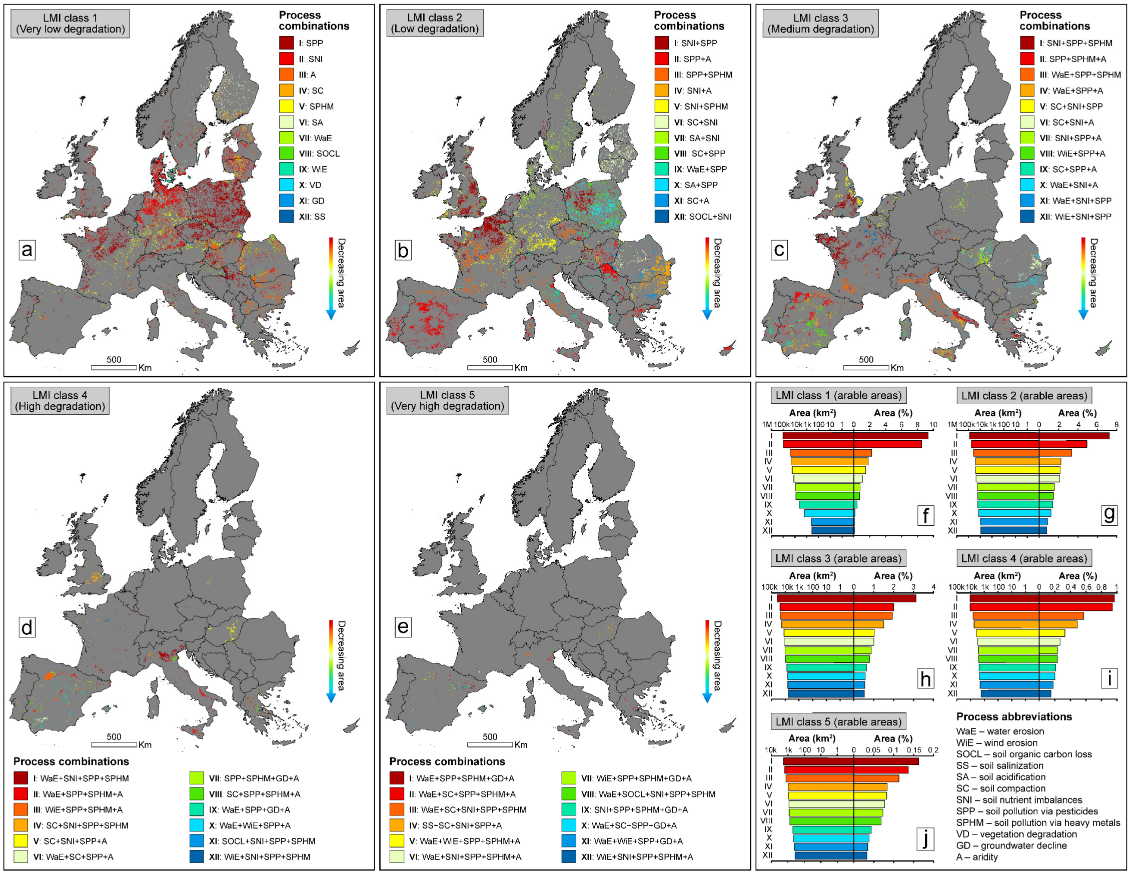


**Supplementary Fig. 4.** Spatial pattern of interacting convergent processes in arable environments of Europe (all 40 investigated countries). **a–e**, Spatial distribution of the dominant (most frequent) co-occurring process types (combinations) in LMI arable classes 1 (a), 2 (b), 3 (c), 4 (d) and 5 (e). **f–j**, Absolute and percentage-based (% of the total continental arable lands) spatial footprint of the dominant co-occurring process types in LMI classes 1 (f), 2 (g), 3 (h), 4 (i) and 5 (j). Notes: in order to simplify the mapping of process combinations (which are very numerous for each LMI class, except for class 1), in this figure the twelve most important types of co-occurring processes in Europe were selected, which, in terms of area, cumulatively account for at least 50% of all LMI class combinations; to better highlight the mapped process combinations, pixel size was increased to 5 km × 5 km, but the quantification of process combination areas (in km$^2$ and %) was done using the original data resolution of 500 m × 500 m; compared to Fig. 4, the process combinations are spatialized here for all analysed states, considering that in some cases with incomplete data (Norway, Switzerland, Balkan countries, Cyprus and Malta) we still used a large number of process databases (9–10 input layers out of 12) in computing LMI (see *Methods*); consequently, LMI still has the potential to provide a good/very good picture of land degradation in these particular countries, even if their LMI combinations should be interpreted with some caution; LMI is the acronym for Land Multi-degradation Index; the source data for the graphs in this figure are provided as a Source Data file.

## Supplementary Tables

**Supplementary Table 1.** Spatial extent (in km$^2$ and %) of LMI classes in agricultural/arable environments of Europe (all 40 investigated countries)

| No. | LMI classes (number of co-occurring processes) | Agricultural lands | | Arable lands | |
|---|---|---|---|---|---|
| | | km$^2$ | % | km$^2$ | % |
| 1 | No degradation (0) [a] | 143,626 | 6.84 | 71,196 | 6.23 |
| 2 | Very low degradation (1) | 577,279 | 27.50 | 304,105 | 26.60 |
| 3 | Low degradation (2) | 727,147 | 34.65 | 395,940 | 34.64 |
| 4 | Medium degradation (3) | 451,213 | 21.50 | 249,178 | 21.80 |
| 5 | High degradation (4) | 156,170 | 7.44 | 95,169 | 8.33 |
| 6 | Very high degradation (≥ 5) [b] | 43,473 | 2.07 | 27,562 | 2.40 |

Notes: a – agricultural/arable lands unaffected by degradation processes; b – most frequently five concurrent processes, according to the LMI histograms for agricultural/arable areas (Supplementary Fig. 2b,e); % – the percentage-based area of the number of convergent processes (0, 1, 2, 3, 4, ≥5), related to the absolute area of continental agricultural (2,098,907 km$^2$)/arable (1,143,149 km$^2$) lands; compared to Table 1, these European statistics are extracted here based on LMI data from all analysed states, considering that in some cases with incomplete data (Norway, Switzerland, Balkan countries, Cyprus and Malta) we still used a large number of process databases (9–10 input layers out of 12) in computing LMI (see *Methods*); consequently, LMI still has the potential to provide a good/very good pan-European picture of land degradation, even if it includes these particular countries, where LMI data should be interpreted with some caution.

**Supplementary Table 2.** Spatial extent (in km$^2$ and %) of agricultural areas and of convergent land degradation processes in agricultural environments of all 40 European countries

| No. [a] | Countries | Land use | LMI classification (number of convergent land degradation processes) | | | | | |
|---|---|---|---|---|---|---|---|---|
| | | Agric. lands (km$^2$) | No degradation | Very low degradation | Low degradation | Medium degradation | High degradation | Very high degradation |
| | | | 0 (%) [b] | 1 (%) | 2 (%) | 3 (%) | 4 (%) | ≥5 (%) [c] |
| 1 | Norway | 15,599 | 40.99 | 30.97 | 19.53 | 7.35 | 1.09 | 0.06 |
| 2 | Finland | 27,555 | 10.64 | 58.70 | 25.78 | 4.60 | 0.27 | 0.01 |
| 3 | Sweden | 39,387 | 20.72 | 37.86 | 32.60 | 8.36 | 0.45 | 0.01 |
| 4 | UK | 136,186 | 3.68 | 18.46 | 37.26 | 29.95 | 9.57 | 1.08 |
| 5 | Estonia | 14,331 | 9.88 | 43.07 | 38.63 | 7.99 | 0.43 | 0.01 |
| 6 | Latvia | 25,624 | 5.13 | 37.65 | 50.25 | 6.72 | 0.24 | 0.01 |
| 7 | Denmark | 31,455 | 19.66 | 50.49 | 23.34 | 5.62 | 0.86 | 0.03 |
| 8 | Lithuania | 38,026 | 17.07 | 49.00 | 30.04 | 3.76 | 0.14 | 0.01 |
| 9 | Ireland | 47,011 | 11.09 | 41.65 | 33.25 | 11.80 | 2.03 | 0.18 |
| 10 | Poland | 183,873 | 3.50 | 45.94 | 39.58 | 9.64 | 1.28 | 0.06 |
| 11 | Germany | 204,657 | 16.81 | 48.01 | 28.32 | 6.22 | 0.62 | 0.02 |
| 12 | Netherlands | 23,710 | 0.43 | 6.32 | 30.80 | 42.08 | 17.09 | 3.28 |
| 13 | Belgium | 17,485 | 1.16 | 12.35 | 50.22 | 26.36 | 8.49 | 1.42 |
| 14 | France | 323,179 | 2.83 | 23.76 | 42.02 | 25.08 | 5.44 | 0.87 |
| 15 | Czechia | 44,715 | 0.71 | 32.60 | 41.94 | 19.95 | 4.48 | 0.33 |
| 16 | Luxembourg | 1373 | 6.83 | 25.09 | 34.10 | 23.43 | 8.70 | 1.84 |
| 17 | Slovakia | 22,942 | 23.65 | 51.39 | 21.33 | 3.42 | 0.20 | 0.01 |
| 18 | Romania | 134,879 | 8.66 | 25.25 | 37.52 | 21.96 | 5.88 | 0.72 |
| 19 | Hungary | 60,556 | 6.09 | 21.22 | 32.87 | 26.21 | 11.04 | 2.58 |
| 20 | Austria | 26,700 | 14.12 | 30.57 | 32.08 | 17.16 | 5.20 | 0.86 |
| 21 | Switzerland | 11,408 | 5.76 | 37.58 | 41.29 | 13.76 | 1.58 | 0.03 |
| 22 | Liechtenstein | 31 | 0.00 | 18.03 | 55.74 | 25.41 | 0.82 | 0.00 |
| 23 | Italy | 156,241 | 1.08 | 8.69 | 27.95 | 38.98 | 19.22 | 4.08 |
| 24 | Slovenia | 6878 | 15.17 | 44.45 | 32.21 | 7.40 | 0.72 | 0.05 |
| 25 | Croatia | 22,131 | 66.18 | 29.66 | 4.00 | 0.15 | <0.01 | 0.00 |
| 26 | Serbia | 42,506 | 1.81 | 41.44 | 48.89 | 7.57 | 0.27 | 0.01 |
| 27 | Bulgaria | 58,104 | 5.23 | 22.97 | 39.26 | 26.48 | 5.52 | 0.55 |
| 28 | BH | 17,141 | 3.36 | 44.30 | 40.61 | 11.42 | 0.30 | 0.00 |
| 29 | Spain | 242,902 | 0.37 | 4.70 | 27.92 | 37.24 | 20.09 | 9.69 |
| 30 | San Marino | 42 | 0.00 | 20.24 | 72.62 | 7.14 | 0.00 | 0.00 |
| 31 | Montenegro | 2182 | 38.62 | 56.50 | 4.81 | 0.07 | 0.00 | 0.00 |
| 32 | Kosovo | 4152 | 0.52 | 26.46 | 55.42 | 16.46 | 1.10 | 0.04 |
| 33 | Monaco | 1 | 66.67 | 33.33 | 0.00 | 0.00 | 0.00 | 0.00 |
| 34 | Portugal | 43,622 | 3.46 | 12.68 | 40.95 | 31.66 | 9.48 | 1.78 |
| 35 | Albania | 7978 | 4.05 | 25.45 | 64.33 | 5.87 | 0.29 | 0.00 |
| 36 | Macedonia | 9131 | 14.20 | 59.83 | 22.57 | 3.35 | 0.04 | 0.00 |
| 37 | Greece | 50,641 | 1.60 | 11.38 | 28.64 | 32.12 | 18.66 | 7.60 |
| 38 | Andorra | 1 | 0.00 | 50.00 | 50.00 | 0.00 | 0.00 | 0.00 |
| 39 | Cyprus | 4422 | 0.79 | 10.96 | 51.73 | 27.67 | 6.52 | 2.33 |
| 40 | Malta | 158 | 0.63 | 11.59 | 39.52 | 38.25 | 9.37 | 0.63 |

Notes: a – the countries are listed in this table (and in the following ones) from north to south, considering the maximum latitude values (in a descending order) of their northern limits; b – agricultural lands unaffected by degradation processes; c – most frequently five co-occurring processes, according to the LMI histogram for agricultural areas (Supplementary Fig. 2b); % – the percentage-based area of the number of convergent processes (0, 1, 2, 3, 4, ≥5), related to the absolute area of national agricultural lands; abbreviations: UK – United Kingdom; BH – Bosnia and Herzegovina; Agric. – Agricultural; LMI statistics should be interpreted with some caution within several European countries (Norway, Switzerland, Balkan countries, Cyprus and Malta), where 9–10 of the 12 input geospatial databases (Fig. 1) were available for LMI computation.

**Supplementary Table 3.** Spatial extent (in $km^2$ and %) of arable areas and of convergent land degradation processes in arable environments of all 40 European countries

| No. | Countries | Land use | LMI classification (number of convergent land degradation processes) | | | | | |
|---|---|---|---|---|---|---|---|---|
| | | Arable lands ($km^2$) | No degradation | Very low degradation | Low degradation | Medium degradation | High degradation | Very high degradation |
| | | | 0 (%) [a] | 1 (%) | 2 (%) | 3 (%) | 4 (%) | ≥5 (%) [b] |
| 1 | Norway | 5601 | 15.71 | 33.11 | 34.93 | 14.23 | 1.92 | 0.11 |
| 2 | Finland | 16,634 | 11.61 | 54.00 | 28.37 | 5.65 | 0.36 | 0.01 |
| 3 | Sweden | 29,875 | 23.35 | 33.84 | 33.25 | 9.04 | 0.50 | 0.01 |
| 4 | UK | 66,324 | 1.53 | 10.65 | 36.91 | 37.07 | 12.68 | 1.16 |
| 5 | Estonia | 6881 | 6.55 | 45.41 | 39.65 | 7.96 | 0.43 | 0.01 |
| 6 | Latvia | 12,121 | 5.33 | 40.37 | 46.69 | 7.25 | 0.37 | 0.01 |
| 7 | Denmark | 26,332 | 17.32 | 51.50 | 24.24 | 5.98 | 0.93 | 0.03 |
| 8 | Lithuania | 22,573 | 16.17 | 46.09 | 33.52 | 4.10 | 0.11 | 0.01 |
| 9 | Ireland | 3171 | 2.66 | 15.15 | 34.34 | 35.20 | 11.26 | 1.39 |
| 10 | Poland | 133,594 | 3.57 | 48.42 | 39.12 | 7.96 | 0.90 | 0.03 |
| 11 | Germany | 135,683 | 17.73 | 50.08 | 26.36 | 5.35 | 0.47 | 0.01 |
| 12 | Netherlands | 7253 | 0.44 | 8.69 | 35.75 | 41.10 | 12.96 | 1.05 |
| 13 | Belgium | 6734 | 0.30 | 6.95 | 60.83 | 24.27 | 6.84 | 0.81 |
| 14 | France | 153,900 | 1.29 | 16.73 | 45.07 | 29.82 | 6.19 | 0.90 |
| 15 | Czechia | 28,736 | 0.38 | 35.26 | 42.65 | 18.00 | 3.47 | 0.24 |
| 16 | Luxembourg | 459 | 6.65 | 28.74 | 33.86 | 21.70 | 7.47 | 1.58 |
| 17 | Slovakia | 15,765 | 25.92 | 52.19 | 18.96 | 2.78 | 0.15 | 0.01 |
| 18 | Romania | 86,225 | 5.39 | 21.63 | 39.75 | 25.40 | 6.99 | 0.84 |
| 19 | Hungary | 47,318 | 6.20 | 20.70 | 32.53 | 26.37 | 11.51 | 2.69 |
| 20 | Austria | 13,005 | 18.61 | 36.49 | 27.15 | 12.73 | 4.40 | 0.62 |
| 21 | Switzerland | 5954 | 2.63 | 32.39 | 46.08 | 16.65 | 2.20 | 0.06 |
| 22 | Liechtenstein | 23 | 0.00 | 16.13 | 59.14 | 24.73 | 0.00 | 0.00 |
| 23 | Italy | 83,054 | 0.65 | 5.25 | 22.55 | 39.35 | 26.34 | 5.86 |
| 24 | Slovenia | 1066 | 20.62 | 37.45 | 30.55 | 10.02 | 1.24 | 0.12 |
| 25 | Croatia | 6180 | 67.10 | 29.19 | 3.53 | 0.16 | 0.02 | 0.00 |
| 26 | Serbia | 21,368 | 1.10 | 26.94 | 63.51 | 8.14 | 0.31 | 0.01 |
| 27 | Bulgaria | 39,186 | 2.06 | 19.45 | 41.14 | 30.17 | 6.51 | 0.67 |
| 28 | BH | 1869 | 4.29 | 40.88 | 39.55 | 14.82 | 0.45 | 0.00 |
| 29 | Spain | 126,868 | 0.18 | 2.35 | 25.29 | 36.00 | 23.43 | 12.76 |
| 30 | San Marino | 20 | 0.00 | 10.00 | 81.25 | 8.75 | 0.00 | 0.00 |
| 31 | Montenegro | 7 | 57.14 | 35.71 | 7.14 | 0.00 | 0.00 | 0.00 |
| 32 | Kosovo | 1279 | 0.27 | 17.54 | 54.04 | 26.00 | 2.09 | 0.06 |
| 33 | Monaco | - | - | - | - | - | - | - |
| 34 | Portugal | 9795 | 1.38 | 11.12 | 42.20 | 32.55 | 9.91 | 2.84 |
| 35 | Albania | 2126 | 6.48 | 38.15 | 45.71 | 8.92 | 0.73 | 0.00 |
| 36 | Macedonia | 2761 | 9.65 | 74.72 | 14.18 | 1.44 | <0.01 | 0.00 |
| 37 | Greece | 20,827 | 0.31 | 7.90 | 30.15 | 34.53 | 20.79 | 6.31 |
| 38 | Andorra | - | - | - | - | - | - | - |
| 39 | Cyprus | 2577 | 0.63 | 7.07 | 56.17 | 26.68 | 5.96 | 3.49 |
| 40 | Malta | 7 | 0.00 | 25.93 | 48.15 | 25.93 | 0.00 | 0.00 |

Notes: a – arable lands unaffected by degradation processes; b – most frequently five co-occurring processes, according to the LMI histogram for arable areas (Supplementary Fig. 2e); % – the percentage-based area of the number of convergent processes (0, 1, 2, 3, 4, ≥5), related to the absolute area of national arable lands; country abbreviations: UK – United Kingdom; BH – Bosnia and Herzegovina; the countries without values (Monaco and Andorra) do not hold any arable lands; LMI statistics should be interpreted with some caution within several European countries (Norway, Switzerland, Balkan countries, Cyprus and Malta), where 9–10 of the 12 input geospatial databases (Fig. 1) were available for LMI computation.

**Supplementary Table 4.** Spatial extent (%) of process types featured in LMI class 1, in agricultural environments of all 40 European countries

| No. | Countries | Area (%) of process types for LMI class 1 (very low degradation) [a] | | | | | | | | | | | |
|---|---|---|---|---|---|---|---|---|---|---|---|---|---|
| | | I | II | III | IV | V | VI | VII | VIII | IX | X | XI | XII |
| 1 | Norway | 9.01 | 6.77 | 0.00 | 0.01 | 0.13 | 0.00 | 3.61 | 4.71 | 0.61 | 5.86 | 0.27 | 0.00 |
| 2 | Finland | 0.03 | 1.41 | 0.00 | 0.00 | 41.47 | 13.79 | 0.01 | 1.26 | 0.53 | 0.18 | 0.01 | 0.00 |
| 3 | Sweden | 0.00 | 16.61 | 0.00 | 0.07 | 14.49 | 0.01 | 0.12 | 3.00 | 2.93 | 0.65 | 0.00 | 0.00 |
| 4 | UK | 3.61 | 10.22 | 1.13 | 0.01 | 1.64 | 0.47 | 0.07 | 0.22 | 0.37 | 0.71 | 0.00 | 0.00 |
| 5 | Estonia | 0.11 | 16.66 | 0.00 | 0.00 | 1.59 | 23.25 | 0.01 | 1.13 | 0.13 | 0.18 | 0.00 | 0.00 |
| 6 | Latvia | 0.04 | 26.62 | 0.00 | 0.00 | 0.53 | 9.68 | 0.01 | 0.49 | 0.14 | 0.14 | 0.00 | 0.00 |
| 7 | Denmark | 0.00 | 32.89 | 0.00 | 0.03 | 3.05 | 2.30 | 0.19 | 1.26 | 10.31 | 0.45 | 0.00 | 0.00 |
| 8 | Lithuania | 0.53 | 31.18 | 0.13 | 0.00 | 1.17 | 14.61 | 0.01 | 0.50 | 0.01 | 0.86 | 0.00 | 0.00 |
| 9 | Ireland | 0.87 | 25.73 | 5.00 | 0.00 | 3.87 | 2.47 | 0.28 | 0.24 | 0.35 | 2.84 | 0.00 | 0.00 |
| 10 | Poland | 42.51 | 0.82 | 0.14 | 0.02 | 0.77 | 1.43 | 0.04 | 0.19 | 0.01 | 0.01 | 0.00 | 0.00 |
| 11 | Germany | 0.03 | 33.39 | 6.44 | 0.37 | 3.26 | 1.19 | 0.44 | 2.67 | 0.11 | 0.12 | 0.00 | 0.00 |
| 12 | Netherlands | 2.76 | 2.81 | 0.06 | 0.00 | 0.10 | 0.41 | 0.00 | 0.11 | 0.05 | 0.01 | 0.01 | 0.00 |
| 13 | Belgium | 10.63 | 0.34 | 0.31 | 0.00 | 0.59 | 0.17 | 0.04 | 0.08 | 0.00 | 0.18 | 0.00 | 0.00 |
| 14 | France | 8.38 | 2.86 | 11.12 | 0.07 | 0.52 | 0.11 | 0.28 | 0.18 | 0.01 | 0.12 | 0.04 | 0.06 |
| 15 | Czechia | 31.17 | 0.71 | 0.14 | 0.09 | 0.36 | 0.00 | 0.05 | 0.08 | 0.00 | 0.00 | 0.00 | 0.00 |
| 16 | Luxembourg | 0.11 | 6.10 | 13.84 | 0.00 | 1.44 | 0.00 | 1.31 | 0.82 | 0.00 | 1.47 | 0.00 | 0.00 |
| 17 | Slovakia | 0.09 | 20.11 | 4.80 | 13.48 | 0.90 | 0.29 | 6.91 | 3.79 | 0.00 | 0.19 | 0.03 | 0.80 |
| 18 | Romania | 0.90 | 6.29 | 0.33 | 6.14 | 0.63 | 6.04 | 3.14 | 1.20 | 0.00 | 0.11 | 0.33 | 0.14 |
| 19 | Hungary | 7.79 | 5.66 | 0.11 | 5.06 | 0.01 | 0.58 | 1.29 | 0.62 | 0.00 | 0.05 | 0.00 | 0.05 |
| 20 | Austria | 0.15 | 5.96 | 6.48 | 4.89 | 4.69 | 0.91 | 5.33 | 2.02 | 0.00 | 0.01 | 0.00 | 0.13 |
| 21 | Switzerland | 31.43 | 2.00 | 0.12 | 0.00 | 0.00 | 0.00 | 2.91 | 0.82 | 0.00 | 0.26 | 0.02 | 0.00 |
| 22 | Liechtenstein | 18.03 | 0.00 | 0.00 | 0.00 | 0.00 | 0.00 | 0.00 | 0.00 | 0.00 | 0.00 | 0.00 | 0.00 |
| 23 | Italy | 4.17 | 0.15 | 1.44 | 2.04 | 0.00 | 0.03 | 0.51 | 0.15 | 0.02 | 0.07 | 0.11 | 0.00 |
| 24 | Slovenia | 0.00 | 3.06 | 27.49 | 0.00 | 2.85 | 2.15 | 6.84 | 1.93 | 0.00 | 0.03 | 0.07 | 0.03 |
| 25 | Croatia | 0.92 | 3.83 | 0.12 | 0.08 | 0.02 | 0.01 | 20.66 | 2.84 | 0.05 | 0.93 | 0.20 | 0.00 |
| 26 | Serbia | 40.13 | 0.11 | 0.00 | 0.72 | 0.00 | 0.00 | 0.40 | 0.04 | 0.00 | 0.00 | 0.04 | 0.00 |
| 27 | Bulgaria | 0.52 | 5.00 | 0.83 | 13.11 | 0.15 | 0.53 | 2.50 | 0.08 | 0.00 | 0.01 | 0.22 | 0.02 |
| 28 | BH | 42.10 | 0.47 | 0.00 | 0.00 | 0.00 | 0.00 | 1.58 | 0.09 | 0.00 | 0.04 | 0.01 | 0.00 |
| 29 | Spain | 0.75 | 0.03 | 1.50 | 2.06 | 0.17 | 0.02 | 0.09 | 0.02 | 0.00 | 0.02 | 0.04 | 0.00 |
| 30 | San Marino | 20.24 | 0.00 | 0.00 | 0.00 | 0.00 | 0.00 | 0.00 | 0.00 | 0.00 | 0.00 | 0.00 | 0.00 |
| 31 | Montenegro | 0.40 | 0.69 | 0.00 | 0.00 | 0.00 | 0.00 | 54.02 | 0.68 | 0.00 | 0.70 | 0.02 | 0.00 |
| 32 | Kosovo | 24.78 | 0.01 | 0.00 | 0.87 | 0.00 | 0.00 | 0.75 | 0.03 | 0.00 | 0.02 | 0.00 | 0.00 |
| 33 | Monaco | 0.00 | 0.00 | 33.33 | 0.00 | 0.00 | 0.00 | 0.00 | 0.00 | 0.00 | 0.00 | 0.00 | 0.00 |
| 34 | Portugal | 7.96 | 0.01 | 0.25 | 3.09 | 0.94 | 0.00 | 0.06 | 0.16 | 0.00 | 0.14 | 0.01 | 0.06 |
| 35 | Albania | 21.34 | 0.24 | 0.00 | 0.02 | 0.00 | 0.00 | 3.74 | 0.02 | 0.00 | 0.09 | 0.01 | 0.00 |
| 36 | Macedonia | 0.46 | 1.44 | 0.00 | 44.44 | 0.00 | 0.00 | 13.13 | 0.25 | 0.00 | 0.09 | 0.03 | 0.00 |
| 37 | Greece | 1.76 | 0.02 | 1.87 | 6.93 | 0.00 | 0.14 | 0.39 | 0.03 | 0.04 | 0.08 | 0.07 | 0.04 |
| 38 | Andorra | 0.00 | 0.00 | 0.00 | 0.00 | 0.00 | 0.00 | 50.00 | 0.00 | 0.00 | 0.00 | 0.00 | 0.00 |
| 39 | Cyprus | 0.45 | 0.00 | 0.00 | 10.04 | 0.00 | 0.01 | 0.03 | 0.01 | 0.40 | 0.02 | 0.01 | 0.00 |
| 40 | Malta | 0.00 | 0.00 | 0.00 | 10.63 | 0.00 | 0.00 | 0.32 | 0.00 | 0.00 | 0.00 | 0.63 | 0.00 |

Notes: a – LMI class 1 strictly includes the twelve processes (I–XII) that act singularly (Supplementary Fig. 3a) and not synergistically in the degradation of agricultural lands: I (SPP), II (SNI), III (SPHM), IV (A), V (SA), VI (SC), VII (WaE), VIII (SOCL), IX (WiE), X (VD), XI (GD), XII (SS); these processes were organised from I to XII according to the descending order of their areas at continental level (Supplementary Fig. 3a,f), which does not necessarily apply nationally; process abbreviations: WaE – water erosion, WiE – wind erosion, SOCL – soil organic carbon loss, SS – soil salinization, SA – soil acidification, SC – soil compaction, SNI – soil nutrient imbalances, SPP – soil pollution via pesticides, SPHM – soil pollution via heavy metals, VD – vegetation degradation, GD – groundwater decline, A – aridity; country abbreviations: UK – United Kingdom, BH – Bosnia and Herzegovina; % – the percentage-based area of the processes, related to the absolute area of the countries' agricultural lands (which can be found in Supplementary Table 2); LMI statistics should be interpreted with some caution within several European countries (Norway, Switzerland, Balkan countries, Cyprus and Malta), where 9–10 of the 12 input geospatial databases (Fig. 1) were available for LMI computation.

**Supplementary Table 5.** Spatial extent (%) of process types (combinations) featured in LMI class 2, in agricultural environments of all 40 European countries

| No. | Countries | Area (%) of 12 major process types for LMI class 2 (low degradation) [a] | | | | | | | | | | | |
|---|---|---|---|---|---|---|---|---|---|---|---|---|---|
| | | I | II | III | IV | V | VI | VII | VIII | IX | X | XI | XII |
| 1 | Norway | 7.68 | 0.00 | 0.00 | 0.00 | 0.00 | 1.29 | 0.01 | 0.00 | 0.00 | 0.00 | 0.00 | 1.96 |
| 2 | Finland | 0.00 | 0.00 | 0.00 | 0.00 | 1.21 | 0.00 | 5.40 | 0.00 | 0.00 | 0.05 | 0.00 | 0.22 |
| 3 | Sweden | 0.00 | 0.00 | 0.00 | 0.01 | 0.00 | 0.00 | 20.27 | 0.01 | 0.00 | 0.00 | 0.00 | 4.78 |
| 4 | UK | 16.99 | 0.00 | 2.34 | 4.80 | 2.05 | 0.11 | 2.28 | 0.01 | 1.54 | 0.55 | 0.00 | 0.30 |
| 5 | Estonia | 0.03 | 0.00 | 0.00 | 0.00 | 29.14 | 0.00 | 1.32 | 0.00 | 0.09 | 0.01 | 0.00 | 2.64 |
| 6 | Latvia | 0.06 | 0.00 | 0.00 | 0.00 | 43.17 | 0.00 | 1.74 | 0.00 | 0.01 | 0.00 | 0.00 | 2.82 |
| 7 | Denmark | 0.00 | 0.00 | 0.00 | 0.04 | 4.50 | 0.00 | 6.08 | 0.00 | 0.00 | 0.00 | 0.00 | 0.92 |
| 8 | Lithuania | 0.37 | 0.00 | 0.00 | 1.05 | 23.13 | 0.00 | 1.27 | 0.00 | 0.27 | 0.03 | 0.00 | 0.65 |
| 9 | Ireland | 3.48 | 0.00 | 0.97 | 14.63 | 1.34 | 0.06 | 0.84 | 0.00 | 0.00 | 0.17 | 0.00 | 0.31 |
| 10 | Poland | 12.65 | 0.38 | 0.16 | 0.01 | 0.26 | 1.35 | 0.13 | 0.03 | 11.10 | 10.53 | 0.00 | 0.04 |
| 11 | Germany | 0.07 | 0.00 | 0.03 | 10.13 | 2.53 | 0.01 | 6.67 | 0.11 | 0.02 | 0.01 | 0.05 | 3.41 |
| 12 | Netherlands | 19.61 | 0.00 | 0.25 | 0.39 | 2.45 | 0.02 | 0.37 | 0.00 | 4.03 | 0.91 | 0.00 | 0.31 |
| 13 | Belgium | 36.49 | 0.00 | 0.40 | 0.19 | 0.06 | 0.21 | 0.46 | 0.00 | 6.80 | 1.39 | 0.00 | 0.02 |
| 14 | France | 13.07 | 0.04 | 13.03 | 7.48 | 0.07 | 0.90 | 0.16 | 0.02 | 0.25 | 0.28 | 0.42 | 0.14 |
| 15 | Czechia | 10.42 | 3.11 | 14.72 | 0.14 | 0.00 | 4.48 | 0.32 | 0.05 | 1.22 | 2.36 | 0.02 | 0.07 |
| 16 | Luxembourg | 0.00 | 0.00 | 0.09 | 10.63 | 0.00 | 0.82 | 0.56 | 0.00 | 0.00 | 0.04 | 0.00 | 0.49 |
| 17 | Slovakia | 0.24 | 0.07 | 0.02 | 3.96 | 0.19 | 2.99 | 2.85 | 3.08 | 0.00 | 0.02 | 0.00 | 2.34 |
| 18 | Romania | 0.41 | 0.30 | 0.01 | 0.22 | 6.20 | 0.69 | 0.35 | 11.80 | 0.39 | 0.03 | 0.03 | 0.48 |
| 19 | Hungary | 6.68 | 10.90 | 0.04 | 0.07 | 0.55 | 0.00 | 0.02 | 4.89 | 1.11 | 0.00 | 0.00 | 0.51 |
| 20 | Austria | 0.04 | 0.06 | 0.04 | 13.15 | 1.30 | 5.74 | 2.03 | 1.52 | 0.01 | 0.01 | 0.48 | 0.68 |
| 21 | Switzerland | 19.05 | 0.00 | 0.15 | 0.10 | 0.00 | 0.01 | 0.00 | 0.00 | 0.01 | 0.01 | 0.00 | 0.29 |
| 22 | Liechtenstein | 28.69 | 0.00 | 0.00 | 0.00 | 0.00 | 4.10 | 0.00 | 0.00 | 0.00 | 0.00 | 0.00 | 0.00 |
| 23 | Italy | 0.99 | 5.96 | 9.53 | 0.26 | 0.00 | 0.00 | 0.00 | 0.20 | 0.24 | 0.00 | 0.79 | 0.02 |
| 24 | Slovenia | 0.00 | 0.00 | 0.17 | 1.78 | 0.25 | 0.00 | 0.83 | 0.00 | 0.00 | 0.00 | 0.00 | 0.36 |
| 25 | Croatia | 0.04 | 0.01 | 0.00 | 0.01 | 0.00 | 0.47 | 0.00 | 0.00 | 0.01 | 0.00 | 0.00 | 0.19 |
| 26 | Serbia | 2.01 | 32.29 | 0.00 | 0.00 | 0.00 | 13.30 | 0.00 | 0.09 | 0.00 | 0.00 | 0.00 | 0.00 |
| 27 | Bulgaria | 0.23 | 0.24 | 0.03 | 1.69 | 0.74 | 0.31 | 0.05 | 17.37 | 0.01 | 0.02 | 0.21 | 0.10 |
| 28 | BH | 6.62 | 0.00 | 0.00 | 0.00 | 0.00 | 31.16 | 0.00 | 0.00 | 0.00 | 0.00 | 0.00 | 0.00 |
| 29 | Spain | 0.03 | 18.33 | 2.38 | 0.16 | 0.00 | 0.70 | 0.01 | 0.05 | 0.00 | 0.21 | 3.15 | 0.00 |
| 30 | San Marino | 0.00 | 0.00 | 1.79 | 0.00 | 0.00 | 70.83 | 0.00 | 0.00 | 0.00 | 0.00 | 0.00 | 0.00 |
| 31 | Montenegro | 0.01 | 0.00 | 0.00 | 0.00 | 0.00 | 1.76 | 0.00 | 0.00 | 0.00 | 0.00 | 0.00 | 0.02 |
| 32 | Kosovo | 0.28 | 14.72 | 0.00 | 0.00 | 0.00 | 39.04 | 0.00 | 0.21 | 0.00 | 0.00 | 0.00 | 0.00 |
| 33 | Monaco | 0.00 | 0.00 | 0.00 | 0.00 | 0.00 | 0.00 | 0.00 | 0.00 | 0.00 | 0.00 | 0.00 | 0.00 |
| 34 | Portugal | 0.20 | 25.94 | 3.45 | 0.00 | 0.00 | 1.74 | 0.01 | 0.11 | 0.00 | 4.03 | 1.56 | 0.01 |
| 35 | Albania | 6.27 | 1.26 | 0.00 | 0.00 | 0.00 | 55.87 | 0.00 | 0.00 | 0.00 | 0.00 | 0.00 | 0.00 |
| 36 | Macedonia | 0.00 | 0.37 | 0.00 | 0.00 | 0.00 | 0.51 | 0.00 | 5.60 | 0.00 | 0.00 | 0.00 | 0.00 |
| 37 | Greece | 0.14 | 11.00 | 4.57 | 0.02 | 0.01 | 1.07 | 0.00 | 0.47 | 0.13 | 0.00 | 5.42 | 0.00 |
| 38 | Andorra | 0.00 | 0.00 | 0.00 | 0.00 | 0.00 | 0.00 | 0.00 | 0.00 | 0.00 | 0.00 | 0.00 | 0.00 |
| 39 | Cyprus | 0.00 | 39.47 | 0.00 | 0.00 | 0.00 | 0.42 | 0.00 | 0.53 | 0.00 | 0.00 | 0.00 | 0.00 |
| 40 | Malta | 0.00 | 0.00 | 0.00 | 0.00 | 0.00 | 0.00 | 0.00 | 0.00 | 0.00 | 0.00 | 0.00 | 0.00 |

Notes: a – in this table, the most important (frequent) twelve types of co-occurring processes (I–XII) in Europe were selected (Supplementary Fig. 3b), which, in terms of area, cumulatively account for at least 50% of the LMI class 2: I (SNI + SPP), II (SPP + A), III (SPP + SPHM), IV (SNI + SPHM), V (SC + SNI), VI (WaE + SPP), VII (SA + SNI), VIII (SNI + A), IX (SC + SPP), X (SA + SPP), XI (SPHM + A), XII (SOCL + SNI); these types (combinations) of processes were organised from I to XII according to the descending order of their areas at continental level (Supplementary Fig. 3b,g), which does not necessarily apply nationally; process abbreviations: WaE – water erosion, SOCL – soil organic carbon loss, SA – soil acidification, SC – soil compaction, SNI – soil nutrient imbalances, SPP – soil pollution via pesticides, SPHM – soil pollution via heavy metals, A – aridity; country abbreviations: UK – United Kingdom, BH – Bosnia and Herzegovina; % – the percentage-based area of the types of convergent processes, related to the absolute area of the countries' agricultural lands (which can be found in Supplementary Table 2); LMI statistics should be interpreted with some caution within several European countries (Norway, Switzerland, Balkan countries, Cyprus and Malta), where 9–10 of the 12 input geospatial databases (Fig. 1) were available for LMI computation.

**Supplementary Table 6.** Spatial extent (%) of process types (combinations) featured in LMI class 3, in agricultural environments of all 40 European countries

| No. [a] | Countries | Area (%) of 12 major process types for LMI class 3 (medium degradation) [b] | | | | | | | | | | | |
|---|---|---|---|---|---|---|---|---|---|---|---|---|---|
| | | I | II | III | IV | V | VI | VII | VIII | IX | X | XI | XII |
| 1 | Norway | 0.00 | 0.00 | 0.00 | 0.00 | 0.00 | 0.00 | 0.00 | 0.00 | 1.01 | 0.00 | 0.00 | 0.00 |
| 2 | Finland | 0.00 | 0.00 | 0.00 | 0.00 | 0.00 | 0.00 | 0.00 | 0.00 | 0.00 | 0.00 | 0.00 | 0.00 |
| 3 | Sweden | 0.00 | 0.00 | 0.00 | 0.00 | 0.00 | 0.00 | 0.00 | 0.00 | 0.00 | 0.00 | 0.00 | 0.00 |
| 4 | UK | 8.92 | 0.01 | 0.08 | 0.00 | 5.64 | 0.00 | 0.01 | 0.00 | 0.72 | 0.00 | 0.00 | 0.00 |
| 5 | Estonia | 0.00 | 0.00 | 0.00 | 0.00 | 0.07 | 0.00 | 0.00 | 0.00 | 0.00 | 0.00 | 0.00 | 0.00 |
| 6 | Latvia | 0.00 | 0.00 | 0.00 | 0.00 | 0.17 | 0.00 | 0.00 | 0.00 | 0.00 | 0.00 | 0.00 | 0.00 |
| 7 | Denmark | 0.00 | 0.00 | 0.00 | 0.00 | 0.00 | 0.00 | 0.00 | 0.00 | 0.00 | 0.00 | 0.00 | 0.00 |
| 8 | Lithuania | 0.00 | 0.00 | 0.00 | 0.00 | 0.15 | 0.00 | 0.00 | 0.00 | 0.00 | 0.00 | 0.00 | 0.00 |
| 9 | Ireland | 4.92 | 0.00 | 0.04 | 0.00 | 0.00 | 0.00 | 0.00 | 0.00 | 0.28 | 0.00 | 0.00 | 0.00 |
| 10 | Poland | 0.01 | 0.00 | 0.01 | 0.00 | 2.89 | 0.00 | 0.67 | 0.03 | 0.09 | 0.45 | 0.00 | 0.00 |
| 11 | Germany | 0.02 | 0.00 | 0.01 | 0.00 | 0.15 | 0.00 | 0.00 | 0.06 | 0.00 | 0.00 | 0.00 | 0.00 |
| 12 | Netherlands | 3.58 | 0.00 | 0.00 | 0.00 | 21.96 | 0.00 | 0.00 | 0.00 | 0.08 | 0.00 | 0.00 | 0.00 |
| 13 | Belgium | 0.33 | 0.00 | 0.01 | 0.00 | 11.75 | 0.00 | 0.00 | 0.00 | 0.58 | 0.00 | 0.00 | 0.00 |
| 14 | France | 10.68 | 0.32 | 3.92 | 0.01 | 0.19 | 0.00 | 0.01 | 0.00 | 0.87 | 0.00 | 0.00 | 0.00 |
| 15 | Czechia | 5.38 | 0.51 | 1.66 | 0.78 | 0.18 | 0.00 | 0.78 | 0.00 | 1.21 | 0.01 | 0.01 | 0.00 |
| 16 | Luxembourg | 0.27 | 0.00 | 0.04 | 0.00 | 0.00 | 0.00 | 0.00 | 0.00 | 0.00 | 0.00 | 0.00 | 0.00 |
| 17 | Slovakia | 0.02 | 0.00 | 0.00 | 0.00 | 0.01 | 0.00 | 0.05 | 0.25 | 0.06 | 0.01 | 0.13 | 0.00 |
| 18 | Romania | 0.01 | 0.00 | 0.00 | 0.19 | 0.21 | 0.00 | 0.10 | 6.86 | 0.18 | 0.12 | 1.72 | 0.01 |
| 19 | Hungary | 0.03 | 0.01 | 0.00 | 0.42 | 0.80 | 0.01 | 11.04 | 2.92 | 1.31 | 4.75 | 0.30 | 0.01 |
| 20 | Austria | 0.04 | 0.04 | 0.01 | 0.01 | 0.03 | 0.00 | 0.04 | 0.04 | 0.01 | 0.00 | 0.35 | 0.00 |
| 21 | Switzerland | 0.12 | 0.00 | 0.08 | 0.00 | 0.00 | 0.00 | 0.00 | 0.00 | 7.27 | 0.00 | 0.00 | 0.00 |
| 22 | Liechtenstein | 0.00 | 0.00 | 0.00 | 0.00 | 0.00 | 0.00 | 0.00 | 0.00 | 0.82 | 0.00 | 0.00 | 0.00 |
| 23 | Italy | 3.02 | 7.88 | 13.64 | 5.13 | 0.06 | 0.75 | 0.39 | 0.00 | 1.66 | 0.51 | 0.15 | 0.46 |
| 24 | Slovenia | 0.00 | 0.00 | 0.11 | 0.00 | 0.00 | 0.00 | 0.00 | 0.00 | 0.00 | 0.00 | 0.00 | 0.00 |
| 25 | Croatia | 0.00 | 0.00 | 0.00 | 0.00 | 0.00 | 0.00 | 0.00 | 0.00 | 0.01 | 0.00 | 0.00 | 0.00 |
| 26 | Serbia | 0.00 | 0.00 | 0.00 | 4.54 | 0.01 | 0.00 | 1.23 | 0.00 | 0.56 | 0.01 | 0.01 | 0.28 |
| 27 | Bulgaria | 0.05 | 0.00 | 0.01 | 0.14 | 0.01 | 0.00 | 0.55 | 3.81 | 0.09 | 0.01 | 10.62 | 0.00 |
| 28 | BH | 0.00 | 0.00 | 0.00 | 0.00 | 0.00 | 0.00 | 0.00 | 0.00 | 9.51 | 0.00 | 0.00 | 0.00 |
| 29 | Spain | 0.11 | 10.53 | 1.50 | 8.42 | 0.00 | 5.37 | 1.21 | 0.01 | 0.01 | 1.51 | 0.01 | 3.05 |
| 30 | San Marino | 0.00 | 0.00 | 6.55 | 0.00 | 0.00 | 0.00 | 0.00 | 0.00 | 0.00 | 0.00 | 0.00 | 0.00 |
| 31 | Montenegro | 0.00 | 0.00 | 0.00 | 0.00 | 0.00 | 0.00 | 0.00 | 0.00 | 0.00 | 0.00 | 0.00 | 0.00 |
| 32 | Kosovo | 0.00 | 0.00 | 0.00 | 12.76 | 0.00 | 0.00 | 0.84 | 0.00 | 0.76 | 0.00 | 0.03 | 0.03 |
| 33 | Monaco | 0.00 | 0.00 | 0.00 | 0.00 | 0.00 | 0.00 | 0.00 | 0.00 | 0.00 | 0.00 | 0.00 | 0.00 |
| 34 | Portugal | 0.06 | 8.54 | 1.43 | 4.06 | 0.00 | 0.32 | 0.85 | 0.02 | 0.03 | 4.58 | 0.01 | 1.72 |
| 35 | Albania | 0.00 | 0.00 | 0.01 | 2.06 | 0.00 | 0.00 | 0.20 | 0.00 | 2.57 | 0.00 | 0.00 | 0.00 |
| 36 | Macedonia | 0.00 | 0.00 | 0.00 | 0.33 | 0.00 | 0.00 | 0.02 | 0.00 | 0.03 | 0.01 | 2.55 | 0.00 |
| 37 | Greece | 0.20 | 9.82 | 4.30 | 4.05 | 0.00 | 0.27 | 1.30 | 0.11 | 0.04 | 1.92 | 0.16 | 0.11 |
| 38 | Andorra | 0.00 | 0.00 | 0.00 | 0.00 | 0.00 | 0.00 | 0.00 | 0.00 | 0.00 | 0.00 | 0.00 | 0.00 |
| 39 | Cyprus | 0.00 | 0.00 | 0.00 | 6.88 | 0.00 | 9.97 | 1.48 | 0.00 | 0.00 | 0.01 | 0.02 | 4.58 |
| 40 | Malta | 0.00 | 0.00 | 0.00 | 0.00 | 0.00 | 0.00 | 0.00 | 0.00 | 0.00 | 0.00 | 0.79 | 0.00 |

Notes: a – in this table, the most important (frequent) twelve types of co-occurring processes (I–XII) in Europe were selected (Supplementary Fig. 3c), which, in terms of area, cumulatively account for at least 50% of the LMI class 3: I (SNI + SPP + SPHM), II (SPP + SPHM + A), III (WaE + SPP + SPHM), IV (WaE + SPP + A), V (SC + SNI + SPP), VI (WiE + SPP + A), VII (SNI + SPP + A), VIII (SC + SNI + A), IX (WaE + SNI + SPP), X (SC + SPP + A), XI (WaE + SNI + A), XII (SPP + GD + A); these types (combinations) of processes were organised from I to XII according to the descending order of their areas at continental level (Supplementary Fig. 3c,h), which does not necessarily apply nationally; process abbreviations: WaE – water erosion, WiE – wind erosion, SC – soil compaction, SNI – soil nutrient imbalances, SPP – soil pollution via pesticides, SPHM – soil pollution via heavy metals, GD – groundwater decline, A – aridity; country abbreviations: UK – United Kingdom, BH – Bosnia and Herzegovina; % – the percentage-based area of the types of convergent processes, related to the absolute area of the countries' agricultural lands (which can be found in Supplementary Table 2); LMI statistics should be interpreted with some caution within several European countries (Norway, Switzerland, Balkan countries, Cyprus and Malta), where 9–10 of the 12 input geospatial databases (Fig. 1) were available for LMI computation.

**Supplementary Table 7.** Spatial extent (%) of process types (combinations) featured in LMI class 4, in agricultural environments of all 40 European countries

| No. | Countries | Area (%) of 12 major process types for LMI class 4 (high degradation) [a] | | | | | | | | | | | |
|---|---|---|---|---|---|---|---|---|---|---|---|---|---|
| | | I | II | III | IV | V | VI | VII | VIII | IX | X | XI | XII |
| 1 | Norway | 0.00 | 0.00 | 0.00 | 0.00 | 0.00 | 0.00 | 0.00 | 0.00 | 0.00 | 0.00 | 0.00 | 0.00 |
| 2 | Finland | 0.00 | 0.00 | 0.00 | 0.00 | 0.00 | 0.00 | 0.00 | 0.00 | 0.00 | 0.00 | 0.00 | 0.00 |
| 3 | Sweden | 0.00 | 0.00 | 0.00 | 0.00 | 0.00 | 0.00 | 0.00 | 0.00 | 0.00 | 0.00 | 0.00 | 0.00 |
| 4 | UK | 0.00 | 0.33 | 3.93 | 0.00 | 0.00 | 0.00 | 0.00 | 0.00 | 0.00 | 0.00 | 0.49 | 0.16 |
| 5 | Estonia | 0.00 | 0.00 | 0.00 | 0.00 | 0.00 | 0.00 | 0.00 | 0.00 | 0.00 | 0.00 | 0.00 | 0.00 |
| 6 | Latvia | 0.00 | 0.00 | 0.00 | 0.00 | 0.00 | 0.00 | 0.00 | 0.00 | 0.00 | 0.00 | 0.00 | 0.00 |
| 7 | Denmark | 0.00 | 0.00 | 0.00 | 0.00 | 0.00 | 0.00 | 0.00 | 0.00 | 0.00 | 0.00 | 0.00 | 0.00 |
| 8 | Lithuania | 0.00 | 0.00 | 0.01 | 0.00 | 0.00 | 0.00 | 0.00 | 0.00 | 0.00 | 0.00 | 0.00 | 0.00 |
| 9 | Ireland | 0.00 | 0.19 | 0.00 | 0.00 | 0.00 | 0.00 | 0.00 | 0.00 | 0.00 | 0.00 | 0.25 | 0.04 |
| 10 | Poland | 0.00 | 0.00 | 0.00 | 0.00 | 0.00 | 0.00 | 0.42 | 0.00 | 0.00 | 0.00 | 0.00 | 0.01 |
| 11 | Germany | 0.00 | 0.01 | 0.00 | 0.00 | 0.00 | 0.00 | 0.00 | 0.00 | 0.00 | 0.00 | 0.00 | 0.01 |
| 12 | Netherlands | 0.00 | 0.00 | 2.93 | 0.00 | 0.00 | 0.00 | 0.00 | 0.00 | 0.00 | 0.00 | 0.25 | 0.71 |
| 13 | Belgium | 0.00 | 0.01 | 1.73 | 0.00 | 0.00 | 0.00 | 0.00 | 0.00 | 0.00 | 0.00 | 0.03 | 0.16 |
| 14 | France | 0.22 | 1.13 | 0.34 | 0.00 | 0.02 | 0.00 | 0.00 | 0.00 | 0.01 | 0.00 | 0.49 | 0.32 |
| 15 | Czechia | 0.07 | 0.51 | 0.03 | 0.00 | 0.00 | 0.00 | 0.00 | 0.00 | 0.00 | 0.00 | 0.60 | 1.60 |
| 16 | Luxembourg | 0.00 | 0.02 | 0.16 | 0.00 | 0.00 | 0.00 | 0.00 | 0.00 | 0.00 | 0.00 | 0.05 | 0.00 |
| 17 | Slovakia | 0.00 | 0.00 | 0.00 | 0.00 | 0.00 | 0.00 | 0.01 | 0.00 | 0.00 | 0.00 | 0.00 | 0.01 |
| 18 | Romania | 0.00 | 0.00 | 0.00 | 0.00 | 0.00 | 0.00 | 0.11 | 0.04 | 0.00 | 0.00 | 0.00 | 0.00 |
| 19 | Hungary | 0.00 | 0.00 | 0.01 | 0.00 | 0.00 | 0.00 | 5.45 | 0.02 | 0.02 | 0.00 | 0.00 | 0.00 |
| 20 | Austria | 0.00 | 0.01 | 0.03 | 0.00 | 0.00 | 0.00 | 0.01 | 0.00 | 0.01 | 0.00 | 0.00 | 0.01 |
| 21 | Switzerland | 0.00 | 0.03 | 0.00 | 0.00 | 0.00 | 0.00 | 0.00 | 0.00 | 0.00 | 0.00 | 0.03 | 0.00 |
| 22 | Liechtenstein | 0.00 | 0.00 | 0.00 | 0.00 | 0.00 | 0.00 | 0.00 | 0.00 | 0.00 | 0.00 | 0.00 | 0.00 |
| 23 | Italy | 3.79 | 6.61 | 0.35 | 0.46 | 0.18 | 0.29 | 0.03 | 0.37 | 0.70 | 0.36 | 0.36 | 0.01 |
| 24 | Slovenia | 0.00 | 0.00 | 0.00 | 0.00 | 0.00 | 0.00 | 0.00 | 0.00 | 0.00 | 0.00 | 0.00 | 0.00 |
| 25 | Croatia | 0.00 | 0.00 | 0.00 | 0.00 | 0.00 | 0.00 | 0.00 | 0.00 | 0.00 | 0.00 | 0.00 | 0.00 |
| 26 | Serbia | 0.00 | 0.00 | 0.00 | 0.00 | 0.00 | 0.01 | 0.01 | 0.00 | 0.00 | 0.00 | 0.00 | 0.00 |
| 27 | Bulgaria | 0.00 | 0.01 | 0.02 | 0.00 | 0.00 | 0.02 | 0.02 | 0.00 | 0.00 | 0.00 | 0.00 | 0.00 |
| 28 | BH | 0.00 | 0.00 | 0.00 | 0.00 | 0.00 | 0.00 | 0.00 | 0.00 | 0.00 | 0.00 | 0.00 | 0.00 |
| 29 | Spain | 2.64 | 0.08 | 0.00 | 2.85 | 2.27 | 1.74 | 0.10 | 1.17 | 0.55 | 1.29 | 0.01 | 0.15 |
| 30 | San Marino | 0.00 | 0.00 | 0.00 | 0.00 | 0.00 | 0.00 | 0.00 | 0.00 | 0.00 | 0.00 | 0.00 | 0.00 |
| 31 | Montenegro | 0.00 | 0.00 | 0.00 | 0.00 | 0.00 | 0.00 | 0.00 | 0.00 | 0.00 | 0.00 | 0.00 | 0.00 |
| 32 | Kosovo | 0.00 | 0.00 | 0.00 | 0.00 | 0.00 | 0.02 | 0.00 | 0.00 | 0.00 | 0.00 | 0.00 | 0.00 |
| 33 | Monaco | 0.00 | 0.00 | 0.00 | 0.00 | 0.00 | 0.00 | 0.00 | 0.00 | 0.00 | 0.00 | 0.00 | 0.00 |
| 34 | Portugal | 1.64 | 0.02 | 0.00 | 0.05 | 0.74 | 0.16 | 0.15 | 0.56 | 0.40 | 0.16 | 0.02 | 0.13 |
| 35 | Albania | 0.00 | 0.00 | 0.00 | 0.00 | 0.00 | 0.00 | 0.00 | 0.00 | 0.00 | 0.00 | 0.00 | 0.00 |
| 36 | Macedonia | 0.00 | 0.00 | 0.00 | 0.00 | 0.00 | 0.00 | 0.00 | 0.00 | 0.00 | 0.00 | 0.00 | 0.00 |
| 37 | Greece | 6.08 | 0.37 | 0.01 | 0.22 | 0.28 | 0.08 | 0.10 | 1.43 | 2.98 | 0.16 | 0.00 | 0.00 |
| 38 | Andorra | 0.00 | 0.00 | 0.00 | 0.00 | 0.00 | 0.00 | 0.00 | 0.00 | 0.00 | 0.00 | 0.00 | 0.00 |
| 39 | Cyprus | 0.00 | 0.00 | 0.00 | 0.00 | 0.00 | 0.21 | 0.01 | 0.00 | 0.00 | 2.53 | 0.00 | 0.00 |
| 40 | Malta | 0.00 | 0.00 | 0.00 | 0.00 | 0.00 | 0.00 | 0.00 | 0.00 | 0.00 | 0.00 | 0.00 | 0.00 |

Notes: a – in this table, the most important (frequent) twelve types of co-occurring processes (I–XII) in Europe were selected (Supplementary Fig. 3d), which, in terms of area, cumulatively account for at least 50% of the LMI class 4: I (WaE + SPP + SPHM + A), II (WaE + SNI + SPP + SPHM), III (SC + SNI + SPP + SPHM), IV (WiE + SPP + SPHM + A), V (SPP + SPHM + GD + A), VI (WaE + SPP + GD + A), VII (SC + SNI + SPP + A), VIII (WaE + SC + SPP + A), IX (SC + SPP + SPHM + A), X (WaE + WiE + SPP + A), XI (SOCL + SNI + SPP + SPHM), XII (SA + SNI + SPP + SPHM); these types (combinations) of processes were organised from I to XII according to the descending order of their areas at continental level (Supplementary Fig. 3d,i), which does not necessarily apply nationally; process abbreviations: WaE – water erosion, WiE – wind erosion, SOCL – soil organic carbon loss, SA – soil acidification, SC – soil compaction, SNI – soil nutrient imbalances, SPP – soil pollution via pesticides, SPHM – soil pollution via heavy metals, GD – groundwater decline, A – aridity; country abbreviations: UK – United Kingdom, BH – Bosnia and Herzegovina; % – the percentage-based area of the types of convergent processes, related to the absolute area of the countries' agricultural lands (which can be found in Supplementary Table 2); LMI statistics should be interpreted with some caution within several European countries (Norway, Switzerland, Balkan countries, Cyprus and Malta), where 9–10 of the 12 input geospatial databases (Fig. 1) were available for LMI computation.

**Supplementary Table 8.** Spatial extent (%) of process types (combinations) featured in LMI class 5, in agricultural environments of all 40 European countries

| No. | Countries | Area (%) of 12 major process types for LMI class 5 (very high degradation) [a] | | | | | | | | | | | |
|---|---|---|---|---|---|---|---|---|---|---|---|---|---|
| | | I | II | III | IV | V | VI | VII | VIII | IX | X | XI | XII |
| 1 | Norway | 0.00 | 0.00 | 0.00 | 0.00 | 0.00 | 0.00 | 0.00 | 0.00 | 0.00 | 0.00 | 0.00 | 0.00 |
| 2 | Finland | 0.00 | 0.00 | 0.00 | 0.00 | 0.00 | 0.00 | 0.00 | 0.00 | 0.00 | 0.00 | 0.00 | 0.00 |
| 3 | Sweden | 0.00 | 0.00 | 0.00 | 0.00 | 0.00 | 0.00 | 0.00 | 0.00 | 0.00 | 0.00 | 0.00 | 0.00 |
| 4 | UK | 0.00 | 0.00 | 0.00 | 0.04 | 0.00 | 0.00 | 0.00 | 0.01 | 0.00 | 0.00 | 0.00 | 0.00 |
| 5 | Estonia | 0.00 | 0.00 | 0.00 | 0.00 | 0.00 | 0.00 | 0.00 | 0.00 | 0.00 | 0.00 | 0.00 | 0.00 |
| 6 | Latvia | 0.00 | 0.00 | 0.00 | 0.00 | 0.00 | 0.00 | 0.00 | 0.00 | 0.00 | 0.00 | 0.00 | 0.00 |
| 7 | Denmark | 0.00 | 0.00 | 0.00 | 0.00 | 0.00 | 0.00 | 0.00 | 0.00 | 0.00 | 0.00 | 0.00 | 0.00 |
| 8 | Lithuania | 0.00 | 0.00 | 0.00 | 0.00 | 0.00 | 0.00 | 0.00 | 0.00 | 0.00 | 0.00 | 0.00 | 0.00 |
| 9 | Ireland | 0.00 | 0.00 | 0.00 | 0.00 | 0.00 | 0.00 | 0.00 | 0.01 | 0.00 | 0.00 | 0.00 | 0.00 |
| 10 | Poland | 0.00 | 0.00 | 0.00 | 0.00 | 0.00 | 0.00 | 0.00 | 0.00 | 0.00 | 0.00 | 0.00 | 0.00 |
| 11 | Germany | 0.00 | 0.00 | 0.00 | 0.00 | 0.00 | 0.00 | 0.00 | 0.00 | 0.00 | 0.00 | 0.00 | 0.00 |
| 12 | Netherlands | 0.00 | 0.00 | 0.00 | 0.00 | 0.00 | 0.00 | 0.00 | 0.00 | 0.00 | 0.00 | 0.00 | 0.00 |
| 13 | Belgium | 0.00 | 0.00 | 0.00 | 0.05 | 0.00 | 0.00 | 0.00 | 0.00 | 0.00 | 0.00 | 0.00 | 0.00 |
| 14 | France | 0.02 | 0.00 | 0.07 | 0.03 | 0.00 | 0.00 | 0.00 | 0.05 | 0.02 | 0.00 | 0.00 | 0.00 |
| 15 | Czechia | 0.00 | 0.00 | 0.01 | 0.00 | 0.00 | 0.00 | 0.00 | 0.05 | 0.00 | 0.00 | 0.00 | 0.00 |
| 16 | Luxembourg | 0.00 | 0.00 | 0.00 | 0.05 | 0.00 | 0.00 | 0.00 | 0.00 | 0.00 | 0.00 | 0.00 | 0.00 |
| 17 | Slovakia | 0.00 | 0.00 | 0.00 | 0.00 | 0.00 | 0.00 | 0.00 | 0.00 | 0.00 | 0.00 | 0.00 | 0.00 |
| 18 | Romania | 0.00 | 0.00 | 0.00 | 0.00 | 0.00 | 0.00 | 0.01 | 0.00 | 0.00 | 0.00 | 0.00 | 0.00 |
| 19 | Hungary | 0.00 | 0.00 | 0.00 | 0.00 | 0.00 | 0.00 | 1.80 | 0.00 | 0.00 | 0.00 | 0.00 | 0.00 |
| 20 | Austria | 0.00 | 0.00 | 0.00 | 0.02 | 0.00 | 0.00 | 0.02 | 0.00 | 0.00 | 0.00 | 0.00 | 0.00 |
| 21 | Switzerland | 0.00 | 0.00 | 0.00 | 0.00 | 0.00 | 0.00 | 0.00 | 0.00 | 0.00 | 0.00 | 0.00 | 0.00 |
| 22 | Liechtenstein | 0.00 | 0.00 | 0.00 | 0.00 | 0.00 | 0.00 | 0.00 | 0.00 | 0.00 | 0.00 | 0.00 | 0.00 |
| 23 | Italy | 0.23 | 0.46 | 0.28 | 0.78 | 0.20 | 0.01 | 0.01 | 0.55 | 0.01 | 0.02 | 0.02 | 0.05 |
| 24 | Slovenia | 0.00 | 0.00 | 0.00 | 0.00 | 0.00 | 0.00 | 0.00 | 0.00 | 0.00 | 0.00 | 0.00 | 0.00 |
| 25 | Croatia | 0.00 | 0.00 | 0.00 | 0.00 | 0.00 | 0.00 | 0.00 | 0.00 | 0.00 | 0.00 | 0.00 | 0.00 |
| 26 | Serbia | 0.00 | 0.00 | 0.00 | 0.00 | 0.00 | 0.00 | 0.00 | 0.00 | 0.00 | 0.00 | 0.00 | 0.00 |
| 27 | Bulgaria | 0.00 | 0.00 | 0.00 | 0.00 | 0.00 | 0.00 | 0.00 | 0.00 | 0.00 | 0.00 | 0.00 | 0.00 |
| 28 | BH | 0.00 | 0.00 | 0.00 | 0.00 | 0.00 | 0.00 | 0.00 | 0.00 | 0.00 | 0.00 | 0.00 | 0.00 |
| 29 | Spain | 1.29 | 0.13 | 0.24 | 0.00 | 0.37 | 0.50 | 0.00 | 0.01 | 0.25 | 0.32 | 0.23 | 0.19 |
| 30 | San Marino | 0.00 | 0.00 | 0.00 | 0.00 | 0.00 | 0.00 | 0.00 | 0.00 | 0.00 | 0.00 | 0.00 | 0.00 |
| 31 | Montenegro | 0.00 | 0.00 | 0.00 | 0.00 | 0.00 | 0.00 | 0.00 | 0.00 | 0.00 | 0.00 | 0.00 | 0.00 |
| 32 | Kosovo | 0.00 | 0.00 | 0.00 | 0.00 | 0.00 | 0.00 | 0.00 | 0.00 | 0.00 | 0.00 | 0.00 | 0.00 |
| 33 | Monaco | 0.00 | 0.00 | 0.00 | 0.00 | 0.00 | 0.00 | 0.00 | 0.00 | 0.00 | 0.00 | 0.00 | 0.00 |
| 34 | Portugal | 0.08 | 0.05 | 0.03 | 0.00 | 0.01 | 0.00 | 0.08 | 0.01 | 0.00 | 0.00 | 0.01 | 0.02 |
| 35 | Albania | 0.00 | 0.00 | 0.00 | 0.00 | 0.00 | 0.00 | 0.00 | 0.00 | 0.00 | 0.00 | 0.00 | 0.00 |
| 36 | Macedonia | 0.00 | 0.00 | 0.00 | 0.00 | 0.00 | 0.00 | 0.00 | 0.00 | 0.00 | 0.00 | 0.00 | 0.00 |
| 37 | Greece | 0.29 | 2.13 | 1.16 | 0.04 | 0.17 | 0.00 | 0.00 | 0.00 | 0.32 | 0.01 | 0.03 | 0.10 |
| 38 | Andorra | 0.00 | 0.00 | 0.00 | 0.00 | 0.00 | 0.00 | 0.00 | 0.00 | 0.00 | 0.00 | 0.00 | 0.00 |
| 39 | Cyprus | 0.00 | 0.00 | 0.00 | 0.00 | 0.00 | 0.00 | 0.03 | 0.00 | 0.00 | 0.11 | 0.00 | 0.01 |
| 40 | Malta | 0.00 | 0.00 | 0.00 | 0.00 | 0.00 | 0.00 | 0.00 | 0.00 | 0.00 | 0.00 | 0.00 | 0.00 |

Notes: a – in this table, the most important (frequent) twelve types of co-occurring processes (I–XII) in Europe were selected (Supplementary Fig. 3e), which, in terms of area, cumulatively account for at least 50% of the LMI class 5: I (WaE + SPP + SPHM + GD + A), II (WaE + SC + SPP + SPHM + A), III (WaE + SNI + SPP + SPHM + A), IV (WaE + SC + SNI + SPP + SPHM), V (WaE + WiE + SPP + SPHM + A), VI (WiE + SPP + SPHM + GD + A), VII (SS + SC + SNI + SPP + A), VIII (WaE + SOCL + SNI + SPP + SPHM), IX (SNI + SPP + SPHM + GD + A), X (WaE + WiE + SPP + GD + A), XI (WaE + SC + SPP + GD + A), XII (WaE + WiE + SNI + SPP + A); these types (combinations) of processes were organised from I to XII according to the descending order of their areas at continental level (Supplementary Fig. 3e,j), which does not necessarily apply nationally; process abbreviations: WaE – water erosion, WiE – wind erosion, SOCL – soil organic carbon loss, SS – soil salinization, SC – soil compaction, SNI – soil nutrient imbalances, SPP – soil pollution via pesticides, SPHM – soil pollution via heavy metals, GD – groundwater decline, A – aridity; country abbreviations: UK – United Kingdom, BH – Bosnia and Herzegovina; % – the percentage-based area of the types of convergent processes, related to the absolute area of the countries' agricultural lands (which can be found in Supplementary Table 2); LMI statistics should be interpreted with some caution within several European countries (Norway, Switzerland, Balkan countries, Cyprus and Malta), where 9–10 of the 12 input geospatial databases (Fig. 1) were available for LMI computation.

**Supplementary Table 9.** Spatial extent (%) of process types featured in LMI class 1, in arable environments of all 40 European countries

| No. | Countries | Area (%) of process types for LMI class 1 (very low degradation) [a] | | | | | | | | | | | |
|---|---|---|---|---|---|---|---|---|---|---|---|---|---|
| | | I | II | III | IV | V | VI | VII | VIII | IX | X | XI | XII |
| 1 | Norway | 9.94 | 16.55 | 0.00 | 0.00 | 0.00 | 0.08 | 1.39 | 2.73 | 0.33 | 2.02 | 0.06 | 0.00 |
| 2 | Finland | 0.03 | 2.10 | 0.00 | 18.54 | 0.00 | 31.00 | 0.01 | 1.62 | 0.53 | 0.17 | 0.01 | 0.00 |
| 3 | Sweden | 0.00 | 19.15 | 0.04 | 0.00 | 0.00 | 7.11 | 0.12 | 3.37 | 3.28 | 0.77 | 0.00 | 0.00 |
| 4 | UK | 2.82 | 6.07 | 0.01 | 0.27 | 0.50 | 0.31 | 0.04 | 0.14 | 0.26 | 0.23 | 0.00 | 0.00 |
| 5 | Estonia | 0.02 | 17.15 | 0.00 | 27.06 | 0.00 | 0.12 | 0.01 | 0.71 | 0.05 | 0.27 | 0.00 | 0.00 |
| 6 | Latvia | 0.01 | 28.47 | 0.00 | 11.05 | 0.00 | 0.08 | 0.01 | 0.34 | 0.18 | 0.22 | 0.00 | 0.00 |
| 7 | Denmark | 0.00 | 34.95 | 0.03 | 1.95 | 0.00 | 2.31 | 0.16 | 1.05 | 10.65 | 0.40 | 0.00 | 0.00 |
| 8 | Lithuania | 0.43 | 24.46 | 0.00 | 19.45 | 0.07 | 0.25 | 0.00 | 0.22 | 0.01 | 1.20 | 0.00 | 0.00 |
| 9 | Ireland | 1.06 | 8.92 | 0.00 | 0.00 | 3.76 | 0.08 | 0.26 | 0.24 | 0.09 | 0.73 | 0.00 | 0.00 |
| 10 | Poland | 45.65 | 0.61 | 0.02 | 1.22 | 0.15 | 0.52 | 0.05 | 0.19 | 0.01 | 0.01 | 0.00 | 0.00 |
| 11 | Germany | 0.02 | 37.96 | 0.40 | 1.21 | 5.43 | 1.97 | 0.43 | 2.47 | 0.12 | 0.08 | 0.00 | 0.00 |
| 12 | Netherlands | 1.81 | 6.60 | 0.00 | 0.07 | 0.00 | 0.01 | 0.00 | 0.06 | 0.12 | 0.02 | 0.00 | 0.00 |
| 13 | Belgium | 6.74 | 0.14 | 0.00 | 0.03 | 0.01 | 0.01 | 0.01 | 0.01 | 0.00 | 0.00 | 0.00 | 0.00 |
| 14 | France | 10.15 | 2.25 | 0.02 | 0.06 | 3.78 | 0.06 | 0.13 | 0.12 | 0.01 | 0.09 | 0.03 | 0.03 |
| 15 | Czechia | 34.77 | 0.17 | 0.12 | 0.00 | 0.07 | 0.04 | 0.04 | 0.05 | 0.00 | 0.00 | 0.00 | 0.00 |
| 16 | Luxembourg | 0.11 | 7.47 | 0.00 | 0.00 | 16.19 | 1.42 | 1.42 | 1.09 | 0.00 | 1.04 | 0.00 | 0.00 |
| 17 | Slovakia | 0.08 | 14.58 | 18.13 | 0.38 | 5.20 | 0.05 | 8.71 | 4.04 | 0.00 | 0.22 | 0.03 | 0.78 |
| 18 | Romania | 0.72 | 3.37 | 7.28 | 6.05 | 0.07 | 0.03 | 2.72 | 0.92 | 0.00 | 0.12 | 0.22 | 0.15 |
| 19 | Hungary | 7.78 | 4.61 | 5.60 | 0.59 | 0.12 | 0.00 | 1.40 | 0.49 | 0.00 | 0.06 | 0.00 | 0.05 |
| 20 | Austria | 0.25 | 6.10 | 9.15 | 1.12 | 6.29 | 2.27 | 8.35 | 2.84 | 0.00 | 0.02 | 0.01 | 0.09 |
| 21 | Switzerland | 28.79 | 1.48 | 0.00 | 0.00 | 0.06 | 0.00 | 1.22 | 0.79 | 0.00 | 0.05 | 0.00 | 0.00 |
| 22 | Liechtenstein | 16.13 | 0.00 | 0.00 | 0.00 | 0.00 | 0.00 | 0.00 | 0.00 | 0.00 | 0.00 | 0.00 | 0.00 |
| 23 | Italy | 2.77 | 0.07 | 1.33 | 0.04 | 0.47 | 0.00 | 0.35 | 0.07 | 0.03 | 0.04 | 0.07 | 0.00 |
| 24 | Slovenia | 0.00 | 11.54 | 0.00 | 2.96 | 8.66 | 3.38 | 7.74 | 3.07 | 0.00 | 0.05 | 0.05 | 0.00 |
| 25 | Croatia | 1.94 | 1.75 | 0.06 | 0.01 | 0.06 | 0.03 | 21.63 | 2.10 | 0.00 | 1.16 | 0.45 | 0.00 |
| 26 | Serbia | 25.65 | 0.15 | 0.93 | 0.00 | 0.00 | 0.00 | 0.14 | 0.02 | 0.00 | 0.00 | 0.06 | 0.00 |
| 27 | Bulgaria | 0.05 | 3.85 | 13.69 | 0.30 | 0.12 | 0.00 | 1.25 | 0.04 | 0.00 | 0.01 | 0.09 | 0.03 |
| 28 | BH | 38.64 | 0.44 | 0.00 | 0.00 | 0.00 | 0.00 | 1.52 | 0.19 | 0.00 | 0.07 | 0.01 | 0.00 |
| 29 | Spain | 0.56 | 0.01 | 1.09 | 0.01 | 0.54 | 0.05 | 0.06 | 0.01 | 0.00 | 0.00 | 0.01 | 0.00 |
| 30 | San Marino | 10.00 | 0.00 | 0.00 | 0.00 | 0.00 | 0.00 | 0.00 | 0.00 | 0.00 | 0.00 | 0.00 | 0.00 |
| 31 | Montenegro | 0.00 | 0.00 | 0.00 | 0.00 | 0.00 | 0.00 | 14.29 | 7.14 | 0.00 | 14.29 | 0.00 | 0.00 |
| 32 | Kosovo | 15.05 | 0.00 | 2.03 | 0.00 | 0.00 | 0.00 | 0.45 | 0.00 | 0.00 | 0.00 | 0.00 | 0.00 |
| 33 | Monaco | - | - | - | - | - | - | - | - | - | - | - | - |
| 34 | Portugal | 6.86 | 0.01 | 3.52 | 0.00 | 0.11 | 0.47 | 0.02 | 0.10 | 0.00 | 0.04 | 0.00 | 0.00 |
| 35 | Albania | 34.27 | 0.31 | 0.07 | 0.00 | 0.00 | 0.00 | 3.35 | 0.01 | 0.00 | 0.13 | 0.01 | 0.00 |
| 36 | Macedonia | 0.13 | 0.27 | 69.35 | 0.00 | 0.00 | 0.00 | 4.70 | 0.16 | 0.00 | 0.11 | 0.00 | 0.00 |
| 37 | Greece | 1.19 | 0.01 | 6.11 | 0.06 | 0.36 | 0.00 | 0.08 | 0.01 | 0.02 | 0.00 | 0.01 | 0.04 |
| 38 | Andorra | - | - | - | - | - | - | - | - | - | - | - | - |
| 39 | Cyprus | 0.12 | 0.00 | 6.55 | 0.00 | 0.00 | 0.00 | 0.01 | 0.01 | 0.37 | 0.01 | 0.01 | 0.00 |
| 40 | Malta | 0.00 | 0.00 | 22.22 | 0.00 | 0.00 | 0.00 | 0.00 | 0.00 | 0.00 | 0.00 | 3.70 | 0.00 |

Notes: a – LMI class 1 strictly includes the twelve processes (I–XII) that act singularly (Supplementary Fig. 4a) and not synergistically in the degradation of arable lands: I (SPP), II (SNI), III (A), IV (SC), V (SPHM), VI (SA), VII (WaE), VIII (SOCL), IX (WiE), X (VD), XI (GD), XII (SS); these processes were organised from I to XII according to the descending order of their areas at continental level (Supplementary Fig. 4a,f), which does not necessarily apply nationally; process abbreviations: WaE – water erosion, WiE – wind erosion, SOCL – soil organic carbon loss, SS – soil salinization, SA – soil acidification, SC – soil compaction, SNI – soil nutrient imbalances, SPP – soil pollution via pesticides, SPHM – soil pollution via heavy metals, VD – vegetation degradation, GD – groundwater decline, A – aridity; country abbreviations: UK – United Kingdom, BH – Bosnia and Herzegovina; % – the percentage-based area of the processes, related to the absolute area of the countries' arable lands (which can be found in Supplementary Table 3); the countries without values (Monaco and Andorra) do not hold any arable lands; LMI statistics should be interpreted with some caution within several European countries (Norway, Switzerland, Balkan countries, Cyprus and Malta), where 9–10 of the 12 input geospatial databases (Fig. 1) were available for LMI computation.

**Supplementary Table 10.** Spatial extent (%) of process types (combinations) featured in LMI class 2, in arable environments of all 40 European countries

| No. | Countries | **Area (%) of 12 major process types for LMI class 2 (low degradation) [a]** | | | | | | | | | | | |
|---|---|---|---|---|---|---|---|---|---|---|---|---|---|
| | | I | II | III | IV | V | VI | VII | VIII | IX | X | XI | XII |
| 1 | Norway | 20.39 | 0.00 | 0.00 | 0.00 | 0.00 | 0.00 | 0.02 | 0.00 | 0.80 | 0.00 | 0.00 | 5.04 |
| 2 | Finland | 0.00 | 0.00 | 0.00 | 0.00 | 0.00 | 1.85 | 7.22 | 0.00 | 0.00 | 0.05 | 0.00 | 0.32 |
| 3 | Sweden | 0.00 | 0.00 | 0.00 | 0.01 | 0.01 | 0.00 | 20.71 | 0.00 | 0.00 | 0.00 | 0.00 | 5.46 |
| 4 | UK | 24.17 | 0.00 | 2.15 | 0.02 | 2.34 | 1.27 | 0.67 | 1.86 | 0.11 | 0.25 | 0.00 | 0.25 |
| 5 | Estonia | 0.00 | 0.00 | 0.00 | 0.00 | 0.00 | 31.29 | 0.82 | 0.08 | 0.00 | 0.00 | 0.00 | 2.43 |
| 6 | Latvia | 0.02 | 0.00 | 0.00 | 0.00 | 0.00 | 40.09 | 0.93 | 0.01 | 0.00 | 0.00 | 0.00 | 2.86 |
| 7 | Denmark | 0.00 | 0.00 | 0.00 | 0.00 | 0.04 | 4.81 | 6.48 | 0.00 | 0.00 | 0.00 | 0.00 | 1.00 |
| 8 | Lithuania | 0.20 | 0.00 | 0.00 | 0.00 | 0.66 | 27.56 | 0.57 | 0.24 | 0.00 | 0.01 | 0.00 | 0.32 |
| 9 | Ireland | 4.72 | 0.00 | 3.41 | 0.00 | 18.95 | 0.01 | 0.20 | 0.00 | 0.06 | 0.01 | 0.00 | 0.60 |
| 10 | Poland | 14.56 | 0.37 | 0.16 | 0.03 | 0.01 | 0.14 | 0.06 | 9.92 | 1.49 | 9.52 | 0.03 | 0.03 |
| 11 | Germany | 0.09 | 0.00 | 0.02 | 0.07 | 10.18 | 2.74 | 5.39 | 0.02 | 0.01 | 0.00 | 0.19 | 3.71 |
| 12 | Netherlands | 27.01 | 0.00 | 0.27 | 0.00 | 0.61 | 1.74 | 0.01 | 1.20 | 0.01 | 0.54 | 0.00 | 0.61 |
| 13 | Belgium | 54.16 | 0.00 | 0.15 | 0.00 | 0.03 | 0.01 | 0.07 | 4.43 | 0.12 | 0.33 | 0.00 | 0.02 |
| 14 | France | 20.28 | 0.01 | 14.97 | 0.00 | 4.55 | 0.03 | 0.05 | 0.21 | 0.97 | 0.13 | 0.00 | 0.13 |
| 15 | Czechia | 9.83 | 4.31 | 14.77 | 0.06 | 0.03 | 0.00 | 0.06 | 1.81 | 5.14 | 1.21 | 0.00 | 0.03 |
| 16 | Luxembourg | 0.00 | 0.00 | 0.11 | 0.00 | 11.12 | 0.00 | 0.55 | 0.00 | 0.00 | 0.00 | 0.00 | 0.60 |
| 17 | Slovakia | 0.16 | 0.10 | 0.02 | 3.80 | 2.34 | 0.21 | 0.96 | 0.00 | 0.06 | 0.00 | 0.52 | 1.75 |
| 18 | Romania | 0.26 | 0.38 | 0.01 | 15.72 | 0.05 | 4.45 | 0.02 | 0.35 | 0.50 | 0.00 | 5.67 | 0.35 |
| 19 | Hungary | 5.59 | 11.61 | 0.04 | 5.14 | 0.06 | 0.42 | 0.01 | 1.06 | 1.67 | 0.00 | 3.61 | 0.31 |
| 20 | Austria | 0.06 | 0.11 | 0.07 | 2.85 | 4.18 | 0.69 | 1.61 | 0.01 | 0.02 | 0.01 | 0.17 | 0.77 |
| 21 | Switzerland | 22.91 | 0.01 | 0.13 | 0.00 | 0.03 | 0.00 | 0.00 | 0.01 | 14.37 | 0.03 | 0.00 | 0.30 |
| 22 | Liechtenstein | 35.48 | 0.00 | 0.00 | 0.00 | 0.00 | 0.00 | 0.00 | 0.00 | 0.00 | 0.00 | 0.00 | 0.00 |
| 23 | Italy | 0.87 | 5.19 | 7.01 | 0.25 | 0.08 | 0.00 | 0.00 | 0.25 | 6.05 | 0.00 | 0.05 | 0.01 |
| 24 | Slovenia | 0.00 | 0.00 | 0.00 | 0.00 | 2.67 | 0.75 | 3.47 | 0.00 | 0.00 | 0.00 | 0.00 | 1.24 |
| 25 | Croatia | 0.09 | 0.04 | 0.00 | 0.00 | 0.02 | 0.00 | 0.00 | 0.01 | 0.99 | 0.00 | 0.00 | 0.06 |
| 26 | Serbia | 2.68 | 52.87 | 0.00 | 0.13 | 0.00 | 0.00 | 0.00 | 0.00 | 6.74 | 0.00 | 0.00 | 0.00 |
| 27 | Bulgaria | 0.15 | 0.13 | 0.01 | 20.20 | 1.07 | 0.52 | 0.01 | 0.00 | 0.02 | 0.00 | 5.30 | 0.08 |
| 28 | BH | 10.11 | 0.00 | 0.00 | 0.00 | 0.00 | 0.00 | 0.00 | 0.00 | 26.22 | 0.00 | 0.00 | 0.00 |
| 29 | Spain | 0.03 | 19.77 | 2.04 | 0.07 | 0.05 | 0.00 | 0.00 | 0.01 | 0.44 | 0.02 | 0.24 | 0.00 |
| 30 | San Marino | 0.00 | 0.00 | 0.00 | 0.00 | 0.00 | 0.00 | 0.00 | 0.00 | 81.25 | 0.00 | 0.00 | 0.00 |
| 31 | Montenegro | 0.00 | 0.00 | 0.00 | 0.00 | 0.00 | 0.00 | 0.00 | 0.00 | 0.00 | 0.00 | 0.00 | 0.00 |
| 32 | Kosovo | 0.31 | 32.34 | 0.00 | 0.55 | 0.00 | 0.00 | 0.00 | 0.00 | 19.77 | 0.00 | 0.00 | 0.00 |
| 33 | Monaco | - | - | - | - | - | - | - | - | - | - | - | - |
| 34 | Portugal | 0.43 | 30.28 | 3.14 | 0.27 | 0.01 | 0.00 | 0.03 | 0.00 | 1.53 | 2.12 | 0.11 | 0.02 |
| 35 | Albania | 14.66 | 2.39 | 0.00 | 0.00 | 0.00 | 0.00 | 0.00 | 0.00 | 27.60 | 0.00 | 0.00 | 0.00 |
| 36 | Macedonia | 0.00 | 0.56 | 0.00 | 3.73 | 0.00 | 0.00 | 0.00 | 0.00 | 0.03 | 0.00 | 0.02 | 0.00 |
| 37 | Greece | 0.18 | 16.04 | 2.21 | 0.54 | 0.02 | 0.01 | 0.00 | 0.04 | 0.69 | 0.00 | 1.53 | 0.00 |
| 38 | Andorra | - | - | - | - | - | - | - | - | - | - | - | - |
| 39 | Cyprus | 0.00 | 46.13 | 0.00 | 0.81 | 0.00 | 0.00 | 0.00 | 0.00 | 0.05 | 0.00 | 0.01 | 0.00 |
| 40 | Malta | 0.00 | 0.00 | 0.00 | 0.00 | 0.00 | 0.00 | 0.00 | 0.00 | 0.00 | 0.00 | 0.00 | 0.00 |

Notes: a – in this table, the most important (frequent) twelve types of co-occurring processes (I–XII) in Europe were selected (Supplementary Fig. 4b), which, in terms of area, cumulatively account for at least 50% of the LMI class 2: I (SNI + SPP), II (SPP + A), III (SPP + SPHM), IV (SNI + A), V (SNI + SPHM), VI (SC + SNI), VII (SA + SNI), VIII (SC + SPP), IX (WaE + SPP), X (SA + SPP), XI (SC + A), XII (SOCL + SNI); these types (combinations) of processes were organised from I to XII according to the descending order of their areas at continental level (Supplementary Fig. 4b,g), which does not necessarily apply nationally; process abbreviations: WaE – water erosion, SOCL – soil organic carbon loss, SA – soil acidification, SC – soil compaction, SNI – soil nutrient imbalances, SPP – soil pollution via pesticides, SPHM – soil pollution via heavy metals, A – aridity; country abbreviations: UK – United Kingdom, BH – Bosnia and Herzegovina; % – the percentage-based area of the types of convergent processes, related to the absolute area of the countries' arable lands (which can be found in Supplementary Table 3); the countries without values (Monaco and Andorra) do not hold any arable lands; LMI statistics should be interpreted with some caution within several European countries (Norway, Switzerland, Balkan countries, Cyprus and Malta), where 9–10 of the 12 input geospatial databases (Fig. 1) were available for LMI computation.

**Supplementary Table 11.** Spatial extent (%) of process types (combinations) featured in LMI class 3, in arable environments of all 40 European countries

| No. | Countries | Area (%) of 12 major process types for LMI class 3 (medium degradation) [a] | | | | | | | | | | | |
|---|---|---|---|---|---|---|---|---|---|---|---|---|---|
| | | I | II | III | IV | V | VI | VII | VIII | IX | X | XI | XII |
| 1 | Norway | 0.00 | 0.00 | 0.00 | 0.00 | 0.00 | 0.00 | 0.00 | 0.00 | 0.00 | 0.00 | 2.29 | 0.24 |
| 2 | Finland | 0.00 | 0.00 | 0.00 | 0.00 | 0.00 | 0.00 | 0.00 | 0.00 | 0.00 | 0.00 | 0.00 | 0.00 |
| 3 | Sweden | 0.00 | 0.00 | 0.00 | 0.00 | 0.00 | 0.00 | 0.00 | 0.00 | 0.00 | 0.00 | 0.00 | 0.00 |
| 4 | UK | 13.32 | 0.01 | 0.07 | 0.00 | 8.57 | 0.01 | 0.03 | 0.00 | 0.00 | 0.00 | 1.08 | 2.58 |
| 5 | Estonia | 0.00 | 0.00 | 0.00 | 0.00 | 0.08 | 0.00 | 0.00 | 0.00 | 0.00 | 0.00 | 0.00 | 0.00 |
| 6 | Latvia | 0.00 | 0.00 | 0.00 | 0.00 | 0.08 | 0.00 | 0.00 | 0.00 | 0.00 | 0.00 | 0.00 | 0.00 |
| 7 | Denmark | 0.00 | 0.00 | 0.00 | 0.00 | 0.00 | 0.00 | 0.00 | 0.00 | 0.00 | 0.00 | 0.00 | 0.00 |
| 8 | Lithuania | 0.00 | 0.00 | 0.00 | 0.00 | 0.08 | 0.00 | 0.00 | 0.00 | 0.00 | 0.00 | 0.00 | 0.00 |
| 9 | Ireland | 21.74 | 0.00 | 0.14 | 0.00 | 0.00 | 0.00 | 0.00 | 0.00 | 0.00 | 0.00 | 0.33 | 0.00 |
| 10 | Poland | 0.01 | 0.00 | 0.01 | 0.00 | 2.20 | 0.01 | 0.81 | 0.00 | 0.32 | 0.00 | 0.09 | 0.11 |
| 11 | Germany | 0.02 | 0.00 | 0.00 | 0.00 | 0.16 | 0.07 | 0.00 | 0.00 | 0.00 | 0.00 | 0.01 | 0.00 |
| 12 | Netherlands | 8.28 | 0.00 | 0.00 | 0.00 | 13.41 | 0.00 | 0.00 | 0.00 | 0.00 | 0.00 | 0.04 | 7.02 |
| 13 | Belgium | 0.22 | 0.00 | 0.01 | 0.00 | 13.95 | 0.00 | 0.00 | 0.00 | 0.00 | 0.00 | 0.76 | 0.10 |
| 14 | France | 13.59 | 0.22 | 4.95 | 0.00 | 0.25 | 0.00 | 0.00 | 0.00 | 0.00 | 0.00 | 1.10 | 2.40 |
| 15 | Czechia | 4.45 | 0.71 | 1.63 | 0.91 | 0.26 | 0.00 | 1.06 | 0.00 | 0.01 | 0.01 | 1.17 | 0.00 |
| 16 | Luxembourg | 0.38 | 0.00 | 0.00 | 0.00 | 0.00 | 0.00 | 0.00 | 0.00 | 0.00 | 0.00 | 0.00 | 0.00 |
| 17 | Slovakia | 0.00 | 0.00 | 0.00 | 0.00 | 0.00 | 0.28 | 0.07 | 0.00 | 0.01 | 0.12 | 0.07 | 0.00 |
| 18 | Romania | 0.00 | 0.00 | 0.00 | 0.20 | 0.18 | 9.28 | 0.13 | 0.00 | 0.14 | 1.74 | 0.14 | 0.00 |
| 19 | Hungary | 0.03 | 0.01 | 0.00 | 0.47 | 0.71 | 3.30 | 11.03 | 0.01 | 5.18 | 0.26 | 1.25 | 0.00 |
| 20 | Austria | 0.03 | 0.09 | 0.01 | 0.02 | 0.03 | 0.08 | 0.08 | 0.00 | 0.00 | 0.52 | 0.00 | 0.00 |
| 21 | Switzerland | 0.14 | 0.00 | 0.10 | 0.00 | 0.00 | 0.00 | 0.00 | 0.00 | 0.00 | 0.00 | 8.53 | 0.00 |
| 22 | Liechtenstein | 0.00 | 0.00 | 0.00 | 0.00 | 0.00 | 0.00 | 0.00 | 0.00 | 0.00 | 0.00 | 1.08 | 0.00 |
| 23 | Italy | 3.81 | 7.06 | 13.08 | 6.37 | 0.11 | 0.00 | 0.49 | 0.76 | 0.61 | 0.17 | 1.80 | 0.02 |
| 24 | Slovenia | 0.00 | 0.00 | 0.00 | 0.00 | 0.00 | 0.00 | 0.00 | 0.00 | 0.00 | 0.00 | 0.00 | 0.00 |
| 25 | Croatia | 0.00 | 0.00 | 0.00 | 0.01 | 0.01 | 0.00 | 0.01 | 0.00 | 0.00 | 0.00 | 0.00 | 0.00 |
| 26 | Serbia | 0.00 | 0.00 | 0.00 | 4.24 | 0.00 | 0.00 | 1.88 | 0.00 | 0.02 | 0.00 | 0.39 | 0.00 |
| 27 | Bulgaria | 0.04 | 0.00 | 0.00 | 0.05 | 0.01 | 4.69 | 0.36 | 0.00 | 0.01 | 11.91 | 0.03 | 0.00 |
| 28 | BH | 0.00 | 0.00 | 0.00 | 0.00 | 0.00 | 0.00 | 0.00 | 0.00 | 0.00 | 0.00 | 12.51 | 0.00 |
| 29 | Spain | 0.05 | 10.72 | 1.93 | 6.50 | 0.00 | 0.01 | 1.37 | 6.28 | 1.84 | 0.01 | 0.01 | 0.00 |
| 30 | San Marino | 0.00 | 0.00 | 8.75 | 0.00 | 0.00 | 0.00 | 0.00 | 0.00 | 0.00 | 0.00 | 0.00 | 0.00 |
| 31 | Montenegro | 0.00 | 0.00 | 0.00 | 0.00 | 0.00 | 0.00 | 0.00 | 0.00 | 0.00 | 0.00 | 0.00 | 0.00 |
| 32 | Kosovo | 0.00 | 0.00 | 0.00 | 19.73 | 0.00 | 0.00 | 2.21 | 0.00 | 0.00 | 0.10 | 0.94 | 0.00 |
| 33 | Monaco | - | - | - | - | - | - | - | - | - | - | - | - |
| 34 | Portugal | 0.14 | 8.42 | 0.72 | 4.39 | 0.00 | 0.00 | 1.56 | 0.20 | 6.96 | 0.01 | 0.02 | 0.00 |
| 35 | Albania | 0.00 | 0.00 | 0.01 | 3.17 | 0.00 | 0.00 | 0.68 | 0.00 | 0.00 | 0.00 | 4.28 | 0.00 |
| 36 | Macedonia | 0.00 | 0.00 | 0.00 | 0.43 | 0.00 | 0.00 | 0.00 | 0.00 | 0.01 | 0.46 | 0.00 | 0.00 |
| 37 | Greece | 0.21 | 10.23 | 2.49 | 5.73 | 0.00 | 0.17 | 1.54 | 0.20 | 3.39 | 0.07 | 0.04 | 0.00 |
| 38 | Andorra | - | - | - | - | - | - | - | - | - | - | - | - |
| 39 | Cyprus | 0.00 | 0.00 | 0.00 | 3.64 | 0.00 | 0.00 | 1.81 | 12.51 | 0.02 | 0.01 | 0.00 | 0.00 |
| 40 | Malta | 0.00 | 0.00 | 0.00 | 0.00 | 0.00 | 0.00 | 0.00 | 0.00 | 0.00 | 3.70 | 0.00 | 0.00 |

Notes: a – in this table, the most important (frequent) twelve types of co-occurring processes (I–XII) in Europe were selected (Supplementary Fig. 4c), which, in terms of area, cumulatively account for at least 50% of the LMI class 3: I (SNI + SPP + SPHM), II (SPP + SPHM + A), III (WaE + SPP + SPHM), IV (WaE + SPP + A), V (SC + SNI + SPP), VI (SC + SNI + A), VII (SNI + SPP + A), VIII (WiE + SPP + A), IX (SC + SPP + A), X (WaE + SNI + A), XI (WaE + SNI + SPP), XII (WiE + SNI + SPP); these types (combinations) of processes were organised from I to XII according to the descending order of their areas at continental level (Supplementary Fig. 4c,h), which does not necessarily apply nationally; process abbreviations: WaE – water erosion, WiE – wind erosion, SC – soil compaction, SNI – soil nutrient imbalances, SPP – soil pollution via pesticides, SPHM – soil pollution via heavy metals, A – aridity; country abbreviations: UK – United Kingdom, BH – Bosnia and Herzegovina; % – the percentage-based area of the types of convergent processes, related to the absolute area of the countries' arable lands (which can be found in Supplementary Table 3); the countries without values (Monaco and Andorra) do not hold any arable lands; LMI statistics should be interpreted with some caution within several European countries (Norway, Switzerland, Balkan countries, Cyprus and Malta), where 9–10 of the 12 input geospatial databases (Fig. 1) were available for LMI computation.

**Supplementary Table 12.** Spatial extent (%) of process types (combinations) featured in LMI class 4, in arable environments of all 40 European countries

| No. | Countries | Area (%) of 12 major process types for LMI class 4 (high degradation) [a] | | | | | | | | | | | |
|---|---|---|---|---|---|---|---|---|---|---|---|---|---|
| | | I | II | III | IV | V | VI | VII | VIII | IX | X | XI | XII |
| 1 | Norway | 0.00 | 0.00 | 0.00 | 0.00 | 0.00 | 0.00 | 0.00 | 0.00 | 0.00 | 0.00 | 0.00 | 0.00 |
| 2 | Finland | 0.00 | 0.00 | 0.00 | 0.00 | 0.00 | 0.00 | 0.00 | 0.00 | 0.00 | 0.00 | 0.00 | 0.00 |
| 3 | Sweden | 0.00 | 0.00 | 0.00 | 0.00 | 0.00 | 0.00 | 0.00 | 0.00 | 0.00 | 0.00 | 0.00 | 0.00 |
| 4 | UK | 0.46 | 0.00 | 0.00 | 6.29 | 0.00 | 0.00 | 0.00 | 0.00 | 0.00 | 0.00 | 0.68 | 1.16 |
| 5 | Estonia | 0.00 | 0.00 | 0.00 | 0.00 | 0.00 | 0.00 | 0.00 | 0.00 | 0.00 | 0.00 | 0.00 | 0.00 |
| 6 | Latvia | 0.00 | 0.00 | 0.00 | 0.00 | 0.00 | 0.00 | 0.00 | 0.00 | 0.00 | 0.00 | 0.00 | 0.00 |
| 7 | Denmark | 0.00 | 0.00 | 0.00 | 0.00 | 0.00 | 0.00 | 0.00 | 0.00 | 0.00 | 0.00 | 0.00 | 0.00 |
| 8 | Lithuania | 0.00 | 0.00 | 0.00 | 0.00 | 0.00 | 0.00 | 0.00 | 0.00 | 0.00 | 0.00 | 0.00 | 0.00 |
| 9 | Ireland | 0.85 | 0.00 | 0.00 | 0.00 | 0.00 | 0.00 | 0.00 | 0.00 | 0.00 | 0.00 | 2.04 | 0.12 |
| 10 | Poland | 0.00 | 0.00 | 0.00 | 0.00 | 0.35 | 0.00 | 0.00 | 0.00 | 0.00 | 0.00 | 0.00 | 0.00 |
| 11 | Germany | 0.01 | 0.00 | 0.00 | 0.00 | 0.00 | 0.00 | 0.00 | 0.00 | 0.00 | 0.00 | 0.00 | 0.00 |
| 12 | Netherlands | 0.00 | 0.00 | 0.00 | 1.02 | 0.00 | 0.00 | 0.00 | 0.00 | 0.00 | 0.00 | 0.60 | 0.77 |
| 13 | Belgium | 0.00 | 0.00 | 0.00 | 1.49 | 0.00 | 0.00 | 0.00 | 0.00 | 0.00 | 0.00 | 0.02 | 0.00 |
| 14 | France | 1.19 | 0.33 | 0.00 | 0.43 | 0.00 | 0.00 | 0.01 | 0.00 | 0.00 | 0.00 | 0.60 | 0.57 |
| 15 | Czechia | 0.40 | 0.06 | 0.00 | 0.04 | 0.00 | 0.00 | 0.00 | 0.00 | 0.00 | 0.00 | 0.52 | 0.00 |
| 16 | Luxembourg | 0.00 | 0.00 | 0.00 | 0.33 | 0.00 | 0.00 | 0.00 | 0.00 | 0.00 | 0.00 | 0.16 | 0.00 |
| 17 | Slovakia | 0.00 | 0.00 | 0.00 | 0.00 | 0.01 | 0.00 | 0.00 | 0.00 | 0.00 | 0.00 | 0.00 | 0.00 |
| 18 | Romania | 0.00 | 0.00 | 0.00 | 0.00 | 0.15 | 0.04 | 0.00 | 0.00 | 0.01 | 0.00 | 0.00 | 0.00 |
| 19 | Hungary | 0.00 | 0.00 | 0.00 | 0.00 | 6.18 | 0.03 | 0.00 | 0.02 | 0.00 | 0.00 | 0.00 | 0.00 |
| 20 | Austria | 0.02 | 0.00 | 0.00 | 0.02 | 0.01 | 0.01 | 0.00 | 0.02 | 0.00 | 0.00 | 0.00 | 0.00 |
| 21 | Switzerland | 0.04 | 0.00 | 0.00 | 0.00 | 0.00 | 0.00 | 0.00 | 0.00 | 0.00 | 0.00 | 0.02 | 0.00 |
| 22 | Liechtenstein | 0.00 | 0.00 | 0.00 | 0.00 | 0.00 | 0.00 | 0.00 | 0.00 | 0.00 | 0.00 | 0.00 | 0.00 |
| 23 | Italy | 10.33 | 5.69 | 0.30 | 0.63 | 0.04 | 0.54 | 0.16 | 1.04 | 0.34 | 0.34 | 0.44 | 0.02 |
| 24 | Slovenia | 0.00 | 0.00 | 0.00 | 0.00 | 0.00 | 0.00 | 0.00 | 0.00 | 0.00 | 0.00 | 0.00 | 0.00 |
| 25 | Croatia | 0.00 | 0.00 | 0.00 | 0.00 | 0.00 | 0.00 | 0.00 | 0.00 | 0.00 | 0.00 | 0.00 | 0.00 |
| 26 | Serbia | 0.00 | 0.00 | 0.00 | 0.00 | 0.02 | 0.00 | 0.00 | 0.00 | 0.02 | 0.00 | 0.00 | 0.00 |
| 27 | Bulgaria | 0.00 | 0.00 | 0.00 | 0.03 | 0.00 | 0.00 | 0.00 | 0.00 | 0.02 | 0.00 | 0.00 | 0.00 |
| 28 | BH | 0.00 | 0.00 | 0.00 | 0.00 | 0.00 | 0.00 | 0.00 | 0.00 | 0.00 | 0.00 | 0.00 | 0.00 |
| 29 | Spain | 0.08 | 3.17 | 4.96 | 0.00 | 0.11 | 1.45 | 1.94 | 0.57 | 1.63 | 1.54 | 0.01 | 0.00 |
| 30 | San Marino | 0.00 | 0.00 | 0.00 | 0.00 | 0.00 | 0.00 | 0.00 | 0.00 | 0.00 | 0.00 | 0.00 | 0.00 |
| 31 | Montenegro | 0.00 | 0.00 | 0.00 | 0.00 | 0.00 | 0.00 | 0.00 | 0.00 | 0.00 | 0.00 | 0.00 | 0.00 |
| 32 | Kosovo | 0.00 | 0.00 | 0.00 | 0.00 | 0.00 | 0.00 | 0.00 | 0.00 | 0.04 | 0.00 | 0.00 | 0.00 |
| 33 | Monaco | - | - | - | - | - | - | - | - | - | - | - | - |
| 34 | Portugal | 0.02 | 1.07 | 0.02 | 0.00 | 0.35 | 0.94 | 0.67 | 0.83 | 0.27 | 0.06 | 0.04 | 0.00 |
| 35 | Albania | 0.00 | 0.00 | 0.00 | 0.00 | 0.00 | 0.00 | 0.00 | 0.00 | 0.00 | 0.00 | 0.00 | 0.00 |
| 36 | Macedonia | 0.00 | 0.00 | 0.00 | 0.00 | 0.00 | 0.01 | 0.00 | 0.00 | 0.00 | 0.00 | 0.00 | 0.00 |
| 37 | Greece | 0.46 | 6.62 | 0.04 | 0.02 | 0.19 | 2.88 | 0.14 | 4.73 | 0.07 | 0.11 | 0.00 | 0.00 |
| 38 | Andorra | - | - | - | - | - | - | - | - | - | - | - | - |
| 39 | Cyprus | 0.00 | 0.00 | 0.00 | 0.00 | 0.01 | 0.00 | 0.00 | 0.00 | 0.15 | 1.24 | 0.00 | 0.00 |
| 40 | Malta | 0.00 | 0.00 | 0.00 | 0.00 | 0.00 | 0.00 | 0.00 | 0.00 | 0.00 | 0.00 | 0.00 | 0.00 |

Notes: a – in this table, the most important (frequent) twelve types of co-occurring processes (I–XII) in Europe were selected (Supplementary Fig. 4d), which, in terms of area, cumulatively account for at least 50% of the LMI class 4: I (WaE + SNI + SPP + SPHM), II (WaE + SPP + SPHM + A), III (WiE + SPP + SPHM + A), IV (SC + SNI + SPP + SPHM), V (SC + SNI + SPP + A), VI (WaE + SC + SPP + A), VII (SPP + SPHM + GD + A), VIII (SC + SPP + SPHM + A), IX (WaE + SPP + GD + A), X (WaE + WiE + SPP + A), XI (SOCL + SNI + SPP + SPHM), XII (WiE + SNI + SPP + SPHM); these types (combinations) of processes were organised from I to XII according to the descending order of their areas at continental level (Supplementary Fig. 4d,i), which does not necessarily apply nationally; process abbreviations: WaE – water erosion, WiE – wind erosion, SOCL – soil organic carbon loss, SC – soil compaction, SNI – soil nutrient imbalances, SPP – soil pollution via pesticides, SPHM – soil pollution via heavy metals, GD – groundwater decline, A – aridity; country abbreviations: UK – United Kingdom, BH – Bosnia and Herzegovina; % – the percentage-based area of the types of convergent processes, related to the absolute area of the countries' arable lands (which can be found in Supplementary Table 3); the countries without values (Monaco and Andorra) do not hold any arable lands; LMI statistics should be interpreted with some caution within several European countries (Norway, Switzerland, Balkan countries, Cyprus and Malta), where 9–10 of the 12 input geospatial databases (Fig. 1) were available for LMI computation.

**Supplementary Table 13.** Spatial extent (%) of process types (combinations) featured in LMI class 5, in arable environments of all 40 European countries

| No. | Countries | Area (%) of 12 major process types for LMI class 5 (very high degradation) [a] | | | | | | | | | | | |
|---|---|---|---|---|---|---|---|---|---|---|---|---|---|
| | | I | II | III | IV | V | VI | VII | VIII | IX | X | XI | XII |
| 1 | Norway | 0.00 | 0.00 | 0.00 | 0.00 | 0.00 | 0.00 | 0.00 | 0.00 | 0.00 | 0.00 | 0.00 | 0.00 |
| 2 | Finland | 0.00 | 0.00 | 0.00 | 0.00 | 0.00 | 0.00 | 0.00 | 0.00 | 0.00 | 0.00 | 0.00 | 0.00 |
| 3 | Sweden | 0.00 | 0.00 | 0.00 | 0.00 | 0.00 | 0.00 | 0.00 | 0.00 | 0.00 | 0.00 | 0.00 | 0.00 |
| 4 | UK | 0.00 | 0.00 | 0.05 | 0.00 | 0.00 | 0.00 | 0.00 | 0.01 | 0.00 | 0.00 | 0.00 | 0.01 |
| 5 | Estonia | 0.00 | 0.00 | 0.00 | 0.00 | 0.00 | 0.00 | 0.00 | 0.00 | 0.00 | 0.00 | 0.00 | 0.00 |
| 6 | Latvia | 0.00 | 0.00 | 0.00 | 0.00 | 0.00 | 0.00 | 0.00 | 0.00 | 0.00 | 0.00 | 0.00 | 0.00 |
| 7 | Denmark | 0.00 | 0.00 | 0.00 | 0.00 | 0.00 | 0.00 | 0.00 | 0.00 | 0.00 | 0.00 | 0.00 | 0.00 |
| 8 | Lithuania | 0.00 | 0.00 | 0.00 | 0.00 | 0.00 | 0.00 | 0.00 | 0.00 | 0.00 | 0.00 | 0.00 | 0.00 |
| 9 | Ireland | 0.00 | 0.00 | 0.00 | 0.00 | 0.00 | 0.00 | 0.00 | 0.14 | 0.00 | 0.00 | 0.00 | 0.00 |
| 10 | Poland | 0.00 | 0.00 | 0.00 | 0.00 | 0.00 | 0.00 | 0.00 | 0.00 | 0.00 | 0.00 | 0.00 | 0.00 |
| 11 | Germany | 0.00 | 0.00 | 0.00 | 0.00 | 0.00 | 0.00 | 0.00 | 0.00 | 0.00 | 0.00 | 0.00 | 0.00 |
| 12 | Netherlands | 0.00 | 0.00 | 0.00 | 0.00 | 0.00 | 0.00 | 0.00 | 0.00 | 0.00 | 0.00 | 0.00 | 0.00 |
| 13 | Belgium | 0.00 | 0.00 | 0.03 | 0.00 | 0.00 | 0.00 | 0.00 | 0.00 | 0.00 | 0.00 | 0.00 | 0.00 |
| 14 | France | 0.02 | 0.00 | 0.04 | 0.00 | 0.00 | 0.05 | 0.00 | 0.04 | 0.01 | 0.00 | 0.00 | 0.00 |
| 15 | Czechia | 0.00 | 0.00 | 0.00 | 0.00 | 0.00 | 0.02 | 0.00 | 0.05 | 0.00 | 0.00 | 0.00 | 0.00 |
| 16 | Luxembourg | 0.00 | 0.00 | 0.11 | 0.00 | 0.00 | 0.00 | 0.00 | 0.00 | 0.00 | 0.00 | 0.00 | 0.00 |
| 17 | Slovakia | 0.00 | 0.00 | 0.00 | 0.00 | 0.00 | 0.00 | 0.00 | 0.00 | 0.00 | 0.00 | 0.00 | 0.00 |
| 18 | Romania | 0.00 | 0.00 | 0.00 | 0.01 | 0.00 | 0.00 | 0.00 | 0.00 | 0.00 | 0.00 | 0.00 | 0.00 |
| 19 | Hungary | 0.00 | 0.00 | 0.00 | 1.90 | 0.00 | 0.00 | 0.00 | 0.00 | 0.00 | 0.00 | 0.00 | 0.00 |
| 20 | Austria | 0.00 | 0.00 | 0.02 | 0.03 | 0.00 | 0.00 | 0.00 | 0.00 | 0.00 | 0.00 | 0.00 | 0.00 |
| 21 | Switzerland | 0.00 | 0.00 | 0.00 | 0.00 | 0.00 | 0.00 | 0.00 | 0.00 | 0.00 | 0.00 | 0.00 | 0.00 |
| 22 | Liechtenstein | 0.00 | 0.00 | 0.00 | 0.00 | 0.00 | 0.00 | 0.00 | 0.00 | 0.00 | 0.00 | 0.00 | 0.00 |
| 23 | Italy | 0.31 | 0.69 | 1.43 | 0.01 | 0.24 | 0.32 | 0.01 | 0.82 | 0.01 | 0.03 | 0.02 | 0.04 |
| 24 | Slovenia | 0.00 | 0.00 | 0.00 | 0.00 | 0.00 | 0.00 | 0.00 | 0.00 | 0.00 | 0.00 | 0.00 | 0.00 |
| 25 | Croatia | 0.00 | 0.00 | 0.00 | 0.00 | 0.00 | 0.00 | 0.00 | 0.00 | 0.00 | 0.00 | 0.00 | 0.00 |
| 26 | Serbia | 0.00 | 0.00 | 0.00 | 0.00 | 0.00 | 0.00 | 0.00 | 0.00 | 0.00 | 0.00 | 0.00 | 0.00 |
| 27 | Bulgaria | 0.00 | 0.00 | 0.00 | 0.00 | 0.00 | 0.00 | 0.00 | 0.00 | 0.00 | 0.00 | 0.00 | 0.00 |
| 28 | BH | 0.00 | 0.00 | 0.00 | 0.00 | 0.00 | 0.00 | 0.00 | 0.00 | 0.00 | 0.00 | 0.00 | 0.00 |
| 29 | Spain | 1.21 | 0.18 | 0.00 | 0.00 | 0.58 | 0.38 | 0.66 | 0.01 | 0.38 | 0.31 | 0.30 | 0.26 |
| 30 | San Marino | 0.00 | 0.00 | 0.00 | 0.00 | 0.00 | 0.00 | 0.00 | 0.00 | 0.00 | 0.00 | 0.00 | 0.00 |
| 31 | Montenegro | 0.00 | 0.00 | 0.00 | 0.00 | 0.00 | 0.00 | 0.00 | 0.00 | 0.00 | 0.00 | 0.00 | 0.00 |
| 32 | Kosovo | 0.00 | 0.00 | 0.00 | 0.00 | 0.00 | 0.00 | 0.00 | 0.00 | 0.00 | 0.00 | 0.00 | 0.00 |
| 33 | Monaco | - | - | - | - | - | - | - | - | - | - | - | - |
| 34 | Portugal | 0.15 | 0.10 | 0.00 | 0.31 | 0.00 | 0.00 | 0.00 | 0.01 | 0.00 | 0.02 | 0.00 | 0.00 |
| 35 | Albania | 0.00 | 0.00 | 0.00 | 0.00 | 0.00 | 0.00 | 0.00 | 0.00 | 0.00 | 0.00 | 0.00 | 0.00 |
| 36 | Macedonia | 0.00 | 0.00 | 0.00 | 0.00 | 0.00 | 0.00 | 0.00 | 0.00 | 0.00 | 0.00 | 0.00 | 0.00 |
| 37 | Greece | 0.08 | 3.57 | 0.07 | 0.00 | 0.03 | 0.18 | 0.00 | 0.00 | 0.01 | 0.05 | 0.00 | 0.00 |
| 38 | Andorra | - | - | - | - | - | - | - | - | - | - | - | - |
| 39 | Cyprus | 0.00 | 0.00 | 0.00 | 0.05 | 0.00 | 0.00 | 0.00 | 0.00 | 0.00 | 0.00 | 0.07 | 0.00 |
| 40 | Malta | 0.00 | 0.00 | 0.00 | 0.00 | 0.00 | 0.00 | 0.00 | 0.00 | 0.00 | 0.00 | 0.00 | 0.00 |

Notes: a – in this table, the most important (frequent) twelve types of co-occurring processes (I–XII) in Europe were selected (Supplementary Fig. 4e), which, in terms of area, cumulatively account for at least 50% of the LMI class 5: I (WaE + SPP + SPHM + GD + A), II (WaE + SC + SPP + SPHM + A), III (WaE + SC + SNI + SPP + SPHM), IV (SS + SC + SNI + SPP + A), V (WaE + WiE + SPP + SPHM + A), VI (WaE + SNI + SPP + SPHM + A), VII (WiE + SPP + SPHM + GD + A), VIII (WaE + SOCL + SNI + SPP + SPHM), IX (SNI + SPP + SPHM + GD + A), X (WaE + SC + SPP + GD + A), XI (WaE + WiE + SPP + GD + A), XII (WiE + SNI + SPP + SPHM + A); these types (combinations) of processes were organised from I to XII according to the descending order of their areas at continental level (Supplementary Fig. 4e,j), which does not necessarily apply nationally; process abbreviations: WaE – water erosion, WiE – wind erosion, SOCL – soil organic carbon loss, SS – soil salinization, SC – soil compaction, SNI – soil nutrient imbalances, SPP – soil pollution via pesticides, SPHM – soil pollution via heavy metals, GD – groundwater decline, A – aridity; country abbreviations: UK – United Kingdom, BH – Bosnia and Herzegovina; % – the percentage-based area of the types of convergent processes, related to the absolute area of the countries' arable lands (which can be found in Supplementary Table 3); the countries without values (Monaco and Andorra) do not hold any arable lands; LMI statistics should be interpreted with some caution within several European countries (Norway, Switzerland, Balkan countries, Cyprus and Malta), where 9–10 of the 12 input geospatial databases (Fig. 1) were available for LMI computation.
