# Supplementary material for: A unifying modelling of multiple land degradation pathways in Europe
Source: Nat Commun. 2024 May 8;15:3862. doi: 10.1038/s41467-024-48252-x (PMC11079025; doi:10.1038/s41467-024-48252-x)
Supplement: Supplementary file 3 — Reporting Summary [file 41467_2024_48252_MOESM3_ESM.pdf]

nature portfolio

Corresponding author(s): Remus Prăvălie

Last updated by author(s): Mar 15, 2024

Reporting Summary

Nature Portfolio wishes to improve the reproducibility of the work that we publish. This form provides structure for consistency and transparency in reporting. For further information on Nature Portfolio policies, see our Editorial Policies and the Editorial Policy Checklist.

Statistics

For all statistical analyses, confirm that the following items are present in the figure legend, table legend, main text, or Methods section.

n/a	Confirmed	
☐	☒	The exact sample size (*n*) for each experimental group/condition, given as a discrete number and unit of measurement
☐	☒	A statement on whether measurements were taken from distinct samples or whether the same sample was measured repeatedly
☐	☒	The statistical test(s) used AND whether they are one- or two-sided<br>*Only common tests should be described solely by name; describe more complex techniques in the Methods section.*
☐	☒	A description of all covariates tested
☐	☒	A description of any assumptions or corrections, such as tests of normality and adjustment for multiple comparisons
☐	☒	A full description of the statistical parameters including central tendency (e.g. means) or other basic estimates (e.g. regression coefficient) AND variation (e.g. standard deviation) or associated estimates of uncertainty (e.g. confidence intervals)
☐	☒	For null hypothesis testing, the test statistic (e.g. *F*, *t*, *r*) with confidence intervals, effect sizes, degrees of freedom and *P* value noted<br>*Give P values as exact values whenever suitable.*
☐	☒	For Bayesian analysis, information on the choice of priors and Markov chain Monte Carlo settings
☐	☒	For hierarchical and complex designs, identification of the appropriate level for tests and full reporting of outcomes
☒	☐	Estimates of effect sizes (e.g. Cohen's *d*, Pearson's *r*), indicating how they were calculated

Our web collection on statistics for biologists contains articles on many of the points above.

Software and code

Policy information about availability of computer code

Data collection	The raw and final databases were collected using R-package (version 1.3-7) and ArcGIS (version 10.5), cited in the paper.
Data analysis	The raw and final databases were modelled, analyzed and mapped using R-package (version 1.3-7), ArcGIS (version 10.5) and Inkscape (version 4), cited in the paper.

For manuscripts utilizing custom algorithms or software that are central to the research but not yet described in published literature, software must be made available to editors and reviewers. We strongly encourage code deposition in a community repository (e.g. GitHub). See the Nature Portfolio guidelines for submitting code & software for further information.

Data

Policy information about availability of data

All manuscripts must include a data availability statement. This statement should provide the following information, where applicable:

- Accession codes, unique identifiers, or web links for publicly available datasets
- A description of any restrictions on data availability
- For clinical datasets or third party data, please ensure that the statement adheres to our policy

The data supporting the findings of this research are available in the article and its Supplementary Information file. Also, the source data for the graphs of the figures are provided as a Source Data file. At the same time, the raster data (GeoTIFF format) of land degradation processes and land multi-degradation in Europe

will be freely available through the European Soil Data Centre (ESDAC), the institutional soil data platform of the European Commission's Joint Research Centre (https://esdac.jrc.ec.europa.eu/). Additional data can be provided by the corresponding author upon reasonable request.

Research involving human participants, their data, or biological material

Policy information about studies with human participants or human data. See also policy information about sex, gender (identity/presentation), and sexual orientation and race, ethnicity and racism.

Reporting on sex and gender	n/a
Reporting on race, ethnicity, or other socially relevant groupings	n/a
Population characteristics	n/a
Recruitment	n/a
Ethics oversight	n/a

Note that full information on the approval of the study protocol must also be provided in the manuscript.

Field-specific reporting

Please select the one below that is the best fit for your research. If you are not sure, read the appropriate sections before making your selection.

☐ Life sciences ☐ Behavioural & social sciences ☒ Ecological, evolutionary & environmental sciences

For a reference copy of the document with all sections, see nature.com/documents/nr-reporting-summary-flat.pdf

Ecological, evolutionary & environmental sciences study design

All studies must disclose on these points even when the disclosure is negative.

Study description	In this study, we present an unprecedented analysis of land multi-degradation in 40 continental countries, using twelve dataset-based processes that were modelled as land degradation convergence and combination pathways in Europe's agricultural (and arable) environments. More specifically, we used a large set of geospatial data (water erosion, wind erosion, soil organic carbon loss, soil salinization, soil acidification, soil compaction, soil nutrient imbalances, soil pollution via pesticides, soil pollution via heavy metals, vegetation degradation, groundwater decline and aridity), which are highly representative for agricultural productivity and that were collected from various sources (n = 6) or developed (n = 6) in this study (see more details in the "Methods" section of the article). We focused the entire analysis on continental (pan-European) agricultural environments, which are critically important for food production, but generally highly vulnerable to multi-degradation. Consequently, here we integrated a complex set of land degradation processes that are strategically important to continental agricultural productivity, thus trying to provide a solid scientific basis for a more realistic and efficient implementation of land degradation-related policies across Europe.
Research sample	We chose the twelve dataset-based processes considering that they are the most relevant for highlighting the agricultural landscapes' degradation in Europe, according to the information documented in literature (and briefly presented in Table 2 of the article). For the selected processes, we collected databases that were already available in their final form for six land degradation processes, the detailed processing information of which can be found directly in some data sources – water erosion, soil organic carbon loss, soil salinization, soil acidification, soil compaction and soil pollution via pesticides (see more details in the "Methods" section of the manuscript). For the other layers, we used various pre-existent data from other sources, in order to refine (wind erosion) or model/obtain (soil nutrient imbalances, soil pollution via heavy metals, vegetation degradation, groundwater decline and aridity) the final data for the remaining six processes (see more details in the "Methods" section of the article). The sources of all databases used are provided in the manuscript (Table 3).
Sampling strategy	All twelve collected/processed spatial databases were finally processed at 500 m (an approximately intermediate spatial resolution in the variety of the original data resolution) and were structured/prepared into 2 general classes, named "Non-critical" and "Critical". The Critical class of each process examined was mapped using critical thresholds documented in literature, over/under which each land degradation process triggers the reduction/loss of agricultural land productivity. The Critical class, which highlights high/severe degradative conditions in agricultural landscapes, was included in the final spatial data modelling, in order to obtain a relevant index for the continental assessment of multiple and convergent land degradation pathways. More specifically, by superimposing/intersecting the 12 datasets, we obtained the Land Multi-degradation Index (LMI), which indicates the simultaneous presence (co-occurrence) of degradative processes, at pixel level.
Data collection	As mentioned above, we collected six databases that were already available in peer-reviewed international scientific literature (water erosion, soil organic carbon loss, soil salinization, soil acidification, soil compaction and soil pollution via pesticides), while for the other six layers (wind erosion, soil nutrient imbalances, soil pollution via heavy metals, vegetation degradation, groundwater decline and aridity) we used various pre-existent data in order to refine or model/obtain the final geospatial data of land degradation processes. Detailed references for all datasets were provided in the article (Table 3).

Timing and spatial scale	The databases used have various temporal and spatial resolutions, according to the technical characteristics specified in Table 3 of the article. The data were finally processed at a standard resolution, for LMI modelling. The spatial scale of the study covers 40 pan-European countries (see more details in the "Methods" section of the article).
Data exclusions	n/a
Reproducibility	The results were presented in the manuscript and in the Supplementary Information file, and the entire analysis is fully reproducible using the geospatial data that will be made freely available on the ESDAC platform of the European Commission.
Randomization	n/a
Blinding	n/a

Did the study involve field work? ☐ Yes ☒ No

Reporting for specific materials, systems and methods

We require information from authors about some types of materials, experimental systems and methods used in many studies. Here, indicate whether each material, system or method listed is relevant to your study. If you are not sure if a list item applies to your research, read the appropriate section before selecting a response.

Materials & experimental systems

n/a	Involved in the study	
☒	☐	Antibodies
☒	☐	Eukaryotic cell lines
☒	☐	Palaeontology and archaeology
☒	☐	Animals and other organisms
☒	☐	Clinical data
☒	☐	Dual use research of concern
☒	☐	Plants

Methods

n/a	Involved in the study	
☒	☐	ChIP-seq
☒	☐	Flow cytometry
☒	☐	MRI-based neuroimaging

Plants

Seed stocks	n/a
Novel plant genotypes	n/a
Authentication	n/a
